# Molecular insights into the promiscuous Ap_4_N hydrolase YqeK

**DOI:** 10.1016/j.jbc.2026.113490

**Published:** 2026-08-25

**Authors:** Renuka Dharani Shivakumar, Natalie Happel, Fabiana Burchert, Michel Brück, Lennart Randau, Jan Pané-Farré, Uwe Linne, Johannes Freitag, Rebecca Hinrichs, Stephan Kiontke, Gert Bange, Patricia Bedrunka-Meinert

**Affiliations:** 1 Center for Synthetic Microbiology (SYNMIKRO), Philipps University, Marburg, Germany; 2 Department of Chemistry, Philipps University, Marburg, Germany; 3 Department of Biology, Philipps University, Marburg, Germany; 4 Microbes-for-Climate (M4C) Cluster of Excellence, Marburg, Germany; 5 Microcosm Earth Center (MEC), Max Planck Institute for Terrestrial Microbiology and Philipps University, Marburg, Germany; 6 Max Planck Institute for terrestrial Microbiology, Marburg, Germany

**Keywords:** hydrolase, alarmone, nucleotide, bacteria, regulation, stress response, biofilm, sporulation

## Abstract

Diadenosine tetraphosphate (Ap_4_A) and related dinucleoside tetraphosphates (Ap_4_Ns) are important stress-signaling molecules that coordinate bacterial adaptation to changing environmental conditions. Although the enzymes for turnover of Ap_4_A are known in several bacteria, the structural basis for substrate recognition and the cellular consequences of impaired Ap_4_A turnover remain poorly understood. Here, we characterize the Histidine-Aspartate (HD)-domain hydrolase YqeK from *Bacillus subtilis*. Deletion of *yqeK* impaired growth in stationary phase, sporulation, and biofilm formation, demonstrating a general role under nutrient limitation. YqeK forms a homodimer and functions as a manganese-dependent phosphohydrolase, symmetrically cleaving Ap_4_A into two ADP molecules and removing Ap_4_A caps from RNA. The enzyme was active not only toward Ap_4_A but also toward the mixed dinucleotides Ap_4_G, Ap_4_C, and Ap_4_U in both *in vitro* and *in vivo* assays, hence acting as a broad-spectrum regulator of Ap_4_N homeostasis. To understand this promiscuity, we determined crystal structures of YqeK in its apo- and ADP-bound state and in complex with a non-hydrolysable Ap_4_A analogue. The structures revealed an asymmetric recognition mechanism in which one nucleoside moiety and the proximal phosphate groups are tightly coordinated, whereas the distal nucleoside is accommodated largely through nonspecific interactions, explaining the ability of YqeK to process diverse substrates. Together, our findings establish YqeK as a central regulator of dinucleotide homeostasis and RNA metabolism and provide the structural framework for Ap_4_N recognition by HD-domain phosphohydrolases.

Diadenosine tetraphosphate (Ap_4_A) is a universal signaling molecule that links cellular stress responses to changes in physiology and survival across diverse organisms (recently reviewed in (1, 2, 3)). Multiple studies have demonstrated that Ap_4_A exerts diverse functions in bacteria, impacting processes such as cell division timing (4), catabolite repression and motility (5), adaptation to heat, oxidative (6, 7) and disulfide stress (8). Further effects of elevated Ap_4_A levels include increased sensitivity to aminoglycoside treatment (9, 10), reduced sporulation (11), and altered pathogenicity and biofilm formation (12, 13, 14, 15).

Ap_4_A is primarily produced by aminoacyl-tRNA synthetases (ARSs), particularly lysyl-tRNA synthetase (*e.g.*, (16, 17)), which catalyze the formation of Ap_4_A through transfer of AMP from the aminoacyl-adenylate intermediate to ATP in the absence of tRNA (18). In addition to Ap_4_A, several ARSs can generate other dinucleoside tetraphosphates, including Ap_4_U, Ap_4_C, and Ap_4_G, through analogous reactions in which UTP, CTP, or GTP, respectively, serve as acceptors in place of ATP. Furthermore, Ap_4_A synthesis is not restricted to ARSs. A variety of additional enzymes, including several ligases and other nucleotide-processing enzymes, have been reported to produce Ap_4_A under specific physiological conditions, highlighting the existence of multiple routes for Ap_4_N formation in the cell (reviewed in: (3)).

Ap_4_A levels are tightly controlled by specific hydrolases and phosphorylases, depending on the respective organism (19): Nudix hydrolases (named for *NUcleoside DIphosphate linked to some other moiety, X*) are conserved across both prokaryotic and eukaryotic organisms (20). They constitute a functionally diverse protein family, acting on a wide range of substrates, including the bacterial alarmones (p)ppGpp and Ap_4_A (20, 21). Ap_4_A-degrading members of the Nudix hydrolases catalyzes the asymmetric cleavage of the molecule, producing ATP and AMP (22, 23, 24). Asymmetric cleavage of Ap_4_A can also occur *via* phosphorolysis, in which inorganic phosphate (P_i_) is used to generate ATP and ADP. In *Saccharomyces cerevisiae*, for example, the phosphorylases Apa1 and Apa2 are able to catalyze this reaction type (25, 26).

Besides the asymmetric cleavage, Ap_4_A can also be symmetrically cleaved by hydrolysis into two ADP molecules. In *Escherichia coli* and other beta- and gamma proteobacteria, this reaction is catalyzed by bis(5′-nucleosyl)-tetraphosphatase ApaH (27). The Gram-positive model organism *Bacillus subtilis* contains the protein phosphatase PrpE, which has a wide substrate spectrum including Ap_4_A (28). Additionally, *B*. *subtilis* harbors the HD-domain family protein YqeK, which can also efficiently cleave Ap_4_A in a symmetric manner (29, 30). Besides its role as an Ap_4_A hydrolase, YqeK also acts as an RNA decapping enzyme; accordingly, *yqeK* deletion in *B. subtilis* elevates Np4 RNA capping and enhances resistance to disulfide stress (8). In *Staphylococcus aureus*, *yqeK* deletion causes massive accumulation of Ap_4_A, impairs resistance to nitrosative and organic acid stress, and reduces virulence gene expression and hemolytic activity, identifying YqeK as a key regulator of stress adaptation and pathogenicity (31).

To further define the physiological role of YqeK, we investigated its contribution to growth, sporulation, and biofilm formation in *B. subtilis*, all of which were affected upon deletion of the corresponding gene. Biochemical analyses demonstrated that YqeK is a manganese-dependent phosphohydrolase that symmetrically cleaves both Ap_4_A and Ap_4_A-capped RNA. It exhibited broad substrate specificity and efficiently hydrolyzed not only Ap_4_A but also the related dinucleoside tetraphosphates Ap_4_U, Ap_4_C, and Ap_4_G. Structural analyses demonstrated that tight coordination of only one of the two nucleoside moieties mechanistically explains this promiscuity. Together, our findings establish YqeK as a multifunctional regulator of nucleotide signaling and RNA metabolism and how HD-domain phosphohydrolases accommodate a broad range of substrates.

## Results

### Deletion of *yqeK* affects growth, sporulation, and biofilm formation

To investigate the physiological role of YqeK in the Gram-positive model organism *B. subtilis*, we constructed a markerless *yqeK* deletion mutant, as well as complementation and overexpression strains, and analyzed growth, sporulation efficiency, biofilm formation, and metabolite/nucleotide profiles. Deletion of *yqeK* did not cause a pronounced growth defect under nutrient-rich conditions. In LB medium, the Δ*yqeK* mutant and the complemented strain only showed a slight reduction in final OD compared with the wild type 168 (WT) (Fig. S1*A*). In contrast, growth in liquid minimal medium revealed a more pronounced phenotype. Compared with the WT strain 168 and the complemented strain, the Δ*yqeK* mutant exhibited a significant defect in late stationary phase, reflected by a marked decrease in optical density (Fig. 1*A*). Consistently, spotting assays of cultures harvested in exponential phase (Fig. S1*B*) and stationary phase (Fig. 1*B*) showed a reduced colony-forming capacity of the Δ*yqeK* mutant on solid minimal medium, indicating impaired survival and/or recovery under nutrient-limiting conditions. Together, these data reveal that YqeK is most likely dispensable under nutrient-rich conditions but is required for optimal fitness during growth in minimal medium, particularly during stationary phase.

**Figure 1:**
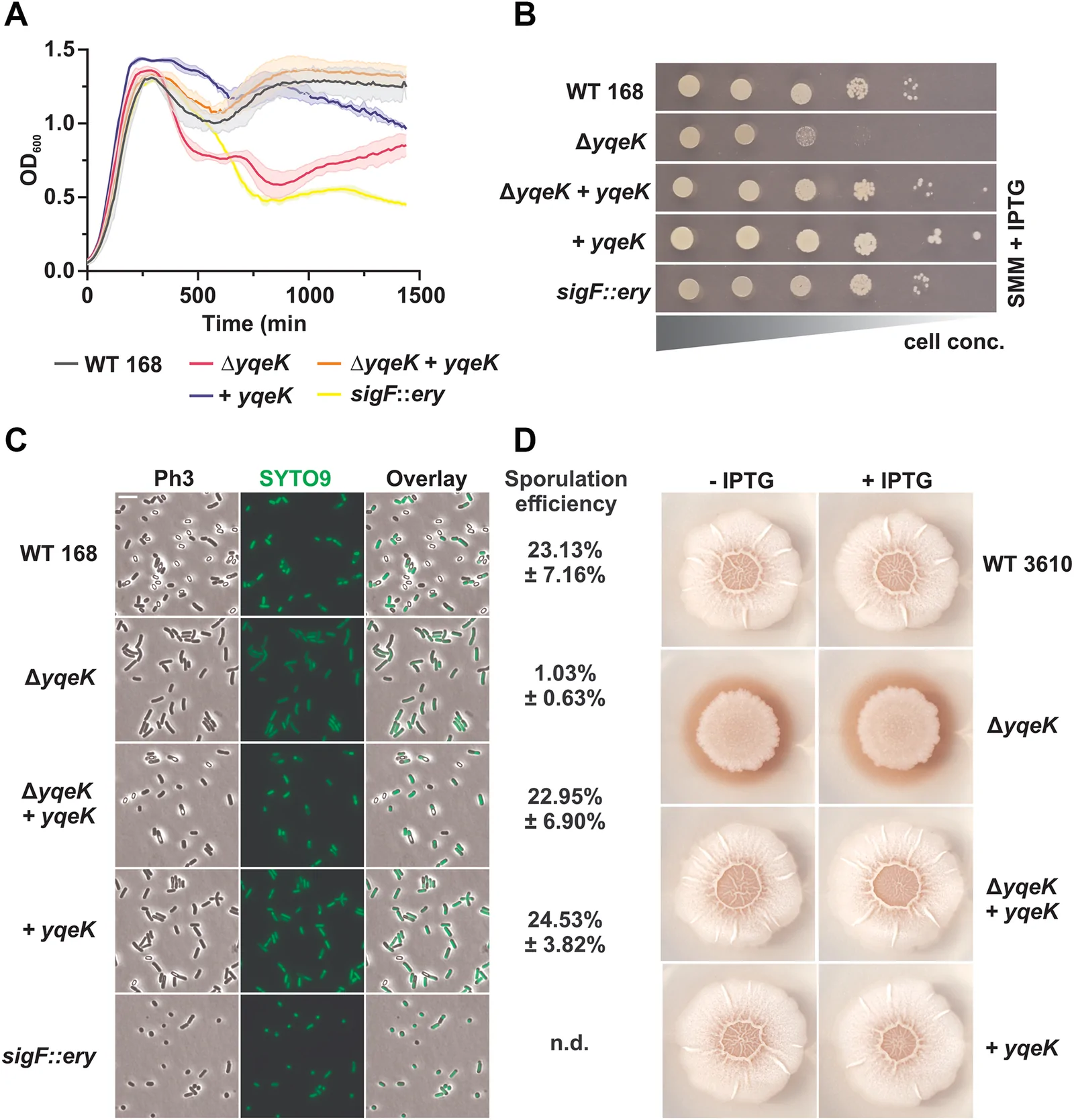
**Deletion of *yqeK* impairs growth, sporulation, and biofilm formation in *B. subtilis*.** Growth curves (*A*) and spot titer assay (*B*) of *B. subtilis* WT 168, the *yqeK* deletion strain [Δ*yqeK* (RS24)], the complementation strain [Δ*yqeK*, *amyE*::*yqeK (ΔyqeK + yqeK*), (RS38)], the overexpression strain [*amyE*::*yqeK (+yqeK*), (RS34)], and the *sigF* deletion strain [Δ*sigF*::*ery* (NH52)]. Optical density at 600 nm (OD_600_) was measured in SMM medium at 37 °C using a plate reader. Error bars represent standard deviations from three technical replicates. For the spot titer assay, cells were grown in SMM at 37 °C to early stationary phase, adjusted to equal optical density, serially diluted, and 3 μl of each dilution were spotted onto solid SMM. (*C*) Shown are phase contrast and fluorescence images of cells (indicated strains) stained with SYTO9 grown for 24 h in SMM medium (Scale bar: 5 μm). (*D*) *B. subtilis* biofilm morphology on MSgg solid medium 72 h post-inoculation in the absence and presence of 1 mM IPTG for wild type strain NCIB3610, the *yqeK* deletion strain Δ*yqeK* (RS25), the complementation strain [Δ*yqeK*, *(ΔyqeK + yqeK*), *amyE*::*yqeK* (RS39)] and overexpression strain [*amyE*::*yqeK (+yqeK*), (RS35)].

The complementation and overexpression strains were generated using the chromosomal integration vector pDR111, in which *yqeK* is expressed from the IPTG-inducible Phy promoter (32, 33). Addition of IPTG did not result in further induction of YqeK, as verified by Western blot analysis of cell extracts using a specific α-YqeK antibody (Fig. S2*B*). This is consistent with the known substantial basal activity of the promoter in the absence of IPTG (34). Consequently, the complemented and overexpression strains overproduce YqeK in the absence of IPTG. This observation applies to all subsequent *in vivo* experiments presented in this study.

Because nutrient limitation is a key trigger for developmental programs in *B. subtilis* (*e.g.*, (35, 36), we next analyzed sporulation efficiency and biofilm formation. In minimal medium, deletion of *yqeK* caused a pronounced defect in sporulation, reducing sporulation efficiency from 23.13 ± 7.2% in the wild type to 1.03 ± 0.6%. This phenotype was fully rescued by complementation with *yqeK*, restoring sporulation efficiency to 22.95 ± 6.9% (Fig. 1*C*). For comparison, we included a *sigF*::*ery* mutant, which lacks the early forespore-specific sigma factor σ^F^ essential for sporulation progression beyond stage II (37). As expected, the *sigF*::*ery* strain formed minicells in minimal medium and displayed reduced growth under these conditions (Fig. 1*A*). However, spotting assays revealed that the *sigF* deletion mutant retained colony-forming capacity comparable to that of the WT (Figs. 1*B* and S1*C*), indicating that its defect primarily affects sporulation rather than general viability in contrast to the Δ*yqeK* mutant. Sporulation assays performed in DSM medium yielded similar results (Fig. S1, *C* and *D*). Whereas the WT strain 168 sporulated with an efficiency of about of approximately 80%, the Δ*yqeK* mutant showed a strong reduction to approximately 20%. Notably, this sporulation defect could not be complemented under these conditions, suggesting that either *yqeK* expression must be precisely controlled or that the sporulation defect depends on a physiological context not fully recapitulated by the complementation strain.

We next assessed the ability of the different strains to form mature biofilms using macro-colony formation on solid MSgg medium (38). As expected and consistent with a previous report (13), deletion of *yqeK* caused a severe defect in macro-colony formation in both the undomesticated NCIB3610 background (Fig. 1*D*) and the laboratory strain 168 (Fig. S1*E*). Whereas WT NCIB3610, complementation, and *yqeK* overexpression strains displayed normal, wrinkled biofilms, the Δ*yqeK* mutant formed flat, less structured colonies lacking the characteristic surface wrinkles. Nonetheless, the Δ*yqeK* biofilm did not resemble that of an, *for example*, *epsH*, *tasA* and *remA* matrix mutants, which are typically completely flat and devoid of any structures (39, 40, 41), indicating that *yqeK* deletion partially, but not completely, disrupts biofilm architecture.

Strain NCIB3610 produces the major biofilm matrix components including EPS and protein-related matrix components. Laboratory strain 168 is defective in robust matrix production due to domestication-associated mutations in matrix-related genes (42, 43). Despite this difference, the *yqeK* deletion displayed similar macro-colony defects in both backgrounds. This finding suggests that the biofilm-associated structural defect caused by *yqeK* deletion is not solely dependent on the presence or absence of major matrix components but rather reflects a more upstream or global effect on developmental programs such as sporulation and biofilm formation. Whereas we could detect clear phenotypes for the *yqeK* deletion, overexpression of *yqeK* in the 168 background revealed only mild physiological effects. These included a prolonged stationary phase (Fig. 1*A*), larger colony-forming units (CFU; Fig. 1*B*), and an increase in macro-colony diameter on solid minimal medium (Fig. S1*E*). Taken together, these results demonstrate that YqeK contributes to multiple developmental and physiological processes in *B. subtilis*, including stationary-phase fitness, sporulation, biofilm formation and potentially motility.

### YqeK symmetrically hydrolyzes Ap_4_A and Ap_4_A-capped RNA in a manganese-dependent manner

A previous study identified YqeK as an Ap_4_A hydrolase (29). To further characterize its biochemical properties, recombinant YqeK proteins from *B. subtilis* (*Bs*YqeK) and *Geobacillus thermodenitrificans* (*Gt*YqeK) were purified to near homogeneity. *Bs*YqeK and *Gt*YqeK exhibit high sequence identity of 68.3% and conservation of the catalytic residues (Fig. S3). Both proteins were purified using a two-step procedure consisting of nickel-affinity chromatography followed by size-exclusion chromatography. For *Bs*YqeK, additional purification steps were introduced, including EDTA treatment and anion-exchange chromatography prior to size-exclusion chromatography, to obtain metal-free protein preparations for subsequent *in vitro* High-Performance Liquid Chromatography (HPLC) and Isothermal Titration Calorimetry (ITC) experiments. For *Gt*YqeK, we also included an EDTA treatment to remove any potentially bound metal ions. In contrast, no EDTA treatment was performed during the purification of the *Gt*YqeK sample used for crystallization (see below). Analytical size-exclusion chromatography showed that both *Bs*- and *Gt*YqeK eluted as a single major species with an apparent molecular mass of approximately 42 kDa, indicating that the proteins predominantly exist as a homodimer in solution (Fig. 2*A*). This is further supported by bacterial hybrid assays performed (Fig. S2*A*). Given that Ap_4_A is a canonical substrate of YqeK, we first confirmed the ability of YqeK to symmetrically hydrolyze Ap_4_A into two ADP molecules by HPLC (Fig. 2*B*). Because YqeK is predicted to be a metal-dependent phosphohydrolase, we tested the effects of several divalent cations, including Mn^2+^, Mg^2+^, Ca^2+^, Cu^2+^, Co^2+^, Zn^2+^, Ni^2+^, and Fe^2+^, on Ap_4_A hydrolysis by EDTA-treated *Bs*YqeK and *Gt*YqeK at metal concentrations of 20 and 500 μM (Fig. S4). For EDTA-treated *Bs*YqeK, Mn^2+^ and Co^2+^ supported extensive and predominantly symmetrical hydrolysis of Ap_4_A to ADP at both concentrations. Fe^2+^ and Cu^2+^ supported hydrolysis but also promoted AMP formation, whereas Ni^2+^ supported only partial hydrolysis at 20 μM Mg^2+^, Ca^2+^, and Zn^2+^ supported little or no activity. EDTA-treated *Gt*YqeK showed a similar metal-dependent activity profile, with Mn^2+^ and Co^2+^ supporting extensive ADP formation, Fe^2+^ and Cu^2+^ producing AMP in addition to ADP, and only limited activity observed with Mg^2+^, Ca^2+^, and Zn^2+^. *Gt*YqeK purified without EDTA treatment was also analyzed for comparison and retained Ap_4_A hydrolysis activity, likely because metal ions acquired during protein production and purification remained bound. Although Co^2+^ supported robust activity *in vitro*, the reported total cell-associated concentrations of Mn^2+^ and Co^2+^ in *B. subtilis* are 26.4 ± 3.1 μM and 0.25 ± 0.15 μM, respectively (44), making Mn^2+^ a physiologically plausible cofactor. Based on its robust and predominantly symmetrical activity across the tested conditions, Mn^2+^ was used as an experimental cofactor in subsequent Ap_4_A hydrolysis assays. Nevertheless, direct determination of the YqeK-bound metal *in vivo* will be required to establish its physiological metalation state.

**Figure 2:**
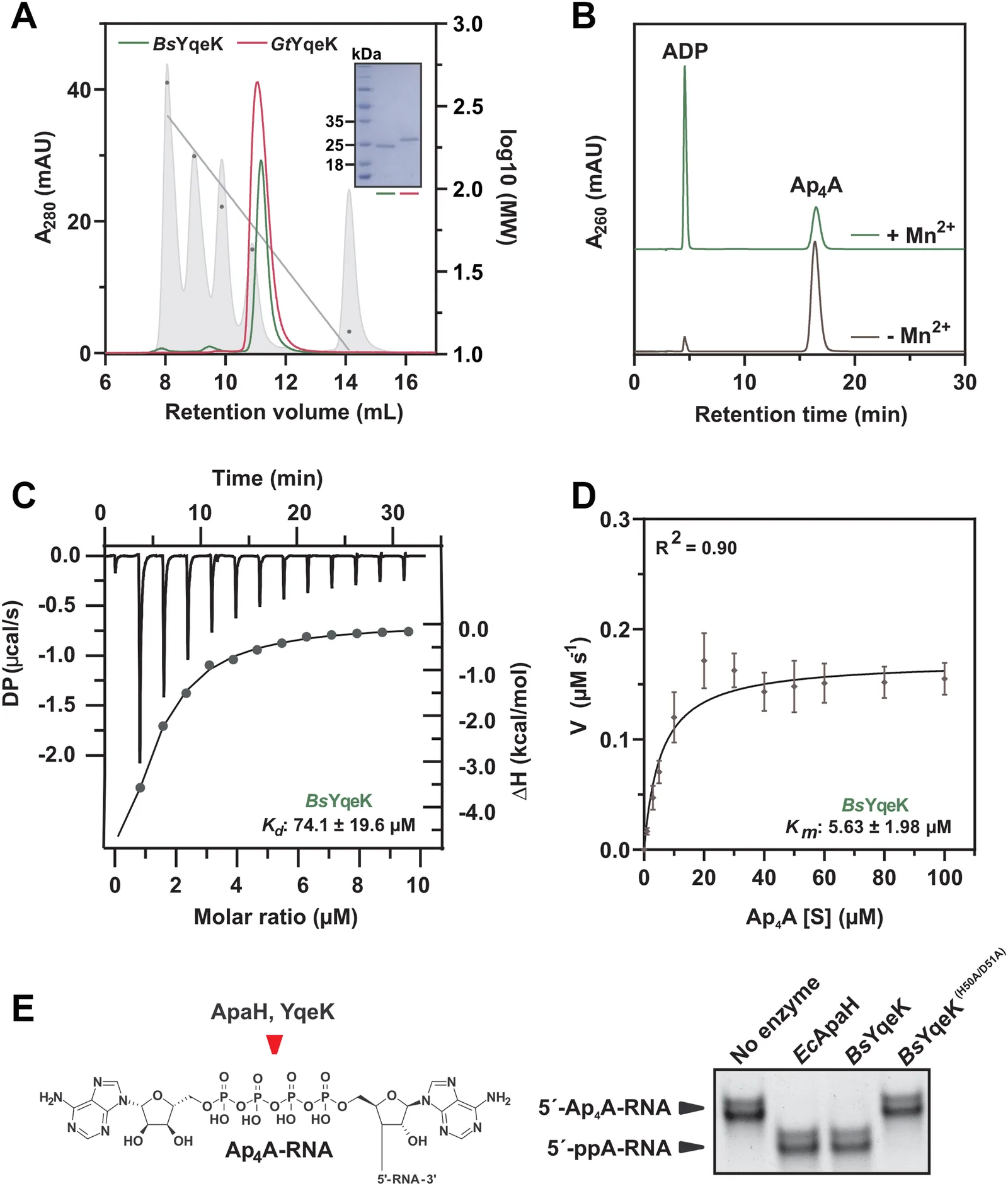
**YqeK is a Ap_4_A hydrolase.***A*, analytical size-exclusion chromatography of purified *Bs*YqeK and *Gt*YqeK. Both proteins elute at approximately 42 kDa, consistent with a homodimer. The *grey* shaded profile and calibration line represent the molecular-weight standards used to estimate the apparent molecular masses. The inset shows the SDS-PAGE of *Bs*YqeK and *Gt*YqeK. *B*, HPLC analysis of Ap_4_A hydrolysis by *Bs*YqeK with or without manganese, which promotes symmetrically converts of Ap_4_A into ADP. *C*, ITC analysis of Ap_4_A binding to *B. subtilis* YqeK. *D*, enzyme-kinetic analysis of *B. subtilis* YqeK mediated hydrolysis of Ap_4_A. A hyperbolic fit according to Michaelis-Menten was used to determine *K*_*m*_ and V_max_. *E*, 5′-Ap_4_A-RNA decapping of *Bs*YqeK *in vitro.* Schematic representation of the 5′-Ap_4_A-RNA decapping reaction (*left*) and analysis of *in vitro* decapping (*right*) of WT *Bs*YqeK and *Bs*YqeK ^(H50A/D51A)^ mutant proteins and the positive control *Ec*ApaH using APB urea polyacrylamide gel electrophoresis.

ITC revealed direct binding of Ap_4_A to *Bs*YqeK with a dissociation constant (*K*_*d*_*)* of 74.1 ± 19.6 μM, indicating moderate substrate affinity (Fig. 2*C*). Because YqeK can hydrolyze Ap_4_A, the ITC signal may include heat contributions from both binding and turnover; therefore, the derived *K*_*d*_ represents an apparent value. We next characterized the catalytic properties of YqeK by steady-state kinetic analysis. Initial reaction velocities increased with increasing substrate concentrations and approached saturation at higher Ap_4_A levels, consistent with Michaelis-Menten kinetics (Fig. 1*D*). Fitting of the data yielded an apparent Michaelis constant (*K*_*m*_) of 5.6 ± 1.9 μM and a maximum velocity (*V*_max_) of 0.20 ± 0.01 μM s^−1^. In this regard, the *K*_*m*_-value of *Bs*YqeK is consistent with that of its *S. aureus* counterpart (*i.e.*, 3.2 μM; (29)), as both enzymes display markedly lower values than those reported for *Streptococcus pneumoniae* SP1746/YqeK (69 μM; (15)) and *Streptococcus pyogenes* YqeK (216 μM; (30)), potentially reflecting species-specific differences in substrate recognition and binding. Altogether, these data demonstrate that *Bs*YqeK is a manganese-dependent Ap_4_A hydrolase that binds its substrate with micromolar affinity and efficiently converts Ap_4_A into ADP through symmetric phosphoanhydride bond cleavage. Besides its role as an Ap_4_A hydrolase, YqeK has recently been reported to function as an RNA decapping enzyme, and deletion of *yqeK* in *B. subtilis* leads to elevated levels of Np4-capped RNAs and increased resistance to disulfide stress (8). To confirm this activity, we generated *in vitro*-transcribed Ap_4_A-capped RNA and incubated it with purified *Bs*YqeK. Analysis of the reaction products demonstrated efficient removal of the Ap_4_A cap by YqeK (Fig. 2*E*). Thus, YqeK functions as a bifunctional hydrolase that targets both free Ap_4_A and Ap_4_A-capped RNA, linking dinucleotide signaling with RNA metabolism.

### *Bs*YqeK hydrolyzes multiple Ap_4_N dinucleotides *in vitro* and *in vivo*

To determine whether *Bs*YqeK is specific for Ap_4_A or capable of hydrolyzing related dinucleoside polyphosphates, we examined its substrate spectrum using a panel of mixed-base Ap_4_N compounds. HPLC analysis revealed manganese-dependent hydrolysis of Ap_4_C, Ap_4_G, and Ap_4_U. In all cases, the reaction products were consistent with symmetric cleavage of the inter-nucleotide polyphosphate chain. Accordingly, Ap_4_A was converted into two ADP molecules, whereas the mixed-base Ap_4_N substrates yielded ADP together with the corresponding nucleoside diphosphate.

To compare substrate preferences, Ap_4_A hydrolysis was defined as 100% relative activity. YqeK hydrolyzed all tested Ap_4_N substrates, albeit with different efficiencies (Table 1; Fig. S5). Among the alternative substrates, Ap_4_G was processed most efficiently, exhibiting a relative activity of 86.1 ± 3.9%. Ap_4_C and Ap_4_U were hydrolyzed less efficiently but still retained substantial activity, with relative activities of 60.9 ± 1.4% and 57.2 ± 3.5%, respectively.

**Table 1:** Relative Activity and Binding Affinity of YqeK Toward Ap_4_N Substrates

Nucleotides	Relative activity (%)	Binding affinity (*K*_*d*_ in μM)
Ap_4_A	100	74.1 ± 19.6
Ap_4_G	86.13 ± 3.94	130 ± 107.6
Ap_4_C	60.94 ± 1.42	33 ± 14.1
Ap_4_U	57.18 ± 3.48	144 ± 64.6

Relative hydrolytic activities of YqeK toward different Ap_4_N dinucleotides, normalized to Ap_4_A (set to 100%). Substrate-binding affinities were determined by isothermal titration calorimetry and are reported as *K*_*d*_ values (μM). Data represent mean ± standard deviation (s.d.) or mean ± fitting error, as appropriate.

To assess substrate recognition, binding affinities were determined by ITC (Table 1; Fig. S6). YqeK bound all tested dinucleotides with micromolar affinity. Ap_4_C and Ap_4_A displayed comparable binding, with *K*_*d*_s of 33.2 ± 14.1 μM and 74.1 ± 19.6 μM, respectively. Binding to Ap_4_G and Ap_4_U was weaker and more variable, with *K*_*d*_ values of 130 ± 107.6 μM and 144 ± 64.6 μM, respectively. Since YqeK can hydrolyze Ap_4_N substrates, the ITC signal may include both binding and turnover heat. Thus, the derived affinities should be interpreted as apparent values, which may partly explain the limited saturation observed for some substrates. Notably, substrate affinity did not strictly correlate with catalytic activity, indicating that efficient turnover depends not only on substrate binding but also on productive positioning within the active site.

To determine whether the broad substrate specificity observed *in vitro* is also relevant *in vivo*, we performed targeted metabolomic analyses of nucleotide extracts from WT 168, Δ*yqeK*, overexpression and the complementation strains. Samples were collected at OD_600_ 0.5, 1.0, 3.0 and after overnight growth (OD_600_ 2.0), and metabolite abundances were normalized to culture density. Consistent with the biochemical data and a previous study showing increased levels of multiple Ap_4_N species in *B. subtilis* cells lacking YqeK (29), deletion of *yqeK* resulted in the significant accumulation of Ap_4_A, Ap_4_G, Ap_4_C, and Ap_4_U across all growth phases (Fig. 3). Among these, Ap_4_A displayed the strongest increase, whereas the remaining Ap_4_N species accumulated to varying extents. Complementation of the mutant restored metabolite levels toward those observed in the wild type, confirming that the observed changes were attributable to the loss of YqeK activity. The accumulation of several structurally distinct Ap_4_N species in the Δ*yqeK* mutant closely mirrors the substrate spectrum determined *in vitro* and demonstrates that YqeK functions as a broad-spectrum dinucleoside polyphosphate hydrolase also *in vivo*. Together, these findings establish YqeK as a key regulator of cellular Ap_4_N homeostasis that is capable of hydrolyzing diverse dinucleoside tetraphosphates differing in both nucleobase composition.

**Figure 3:**
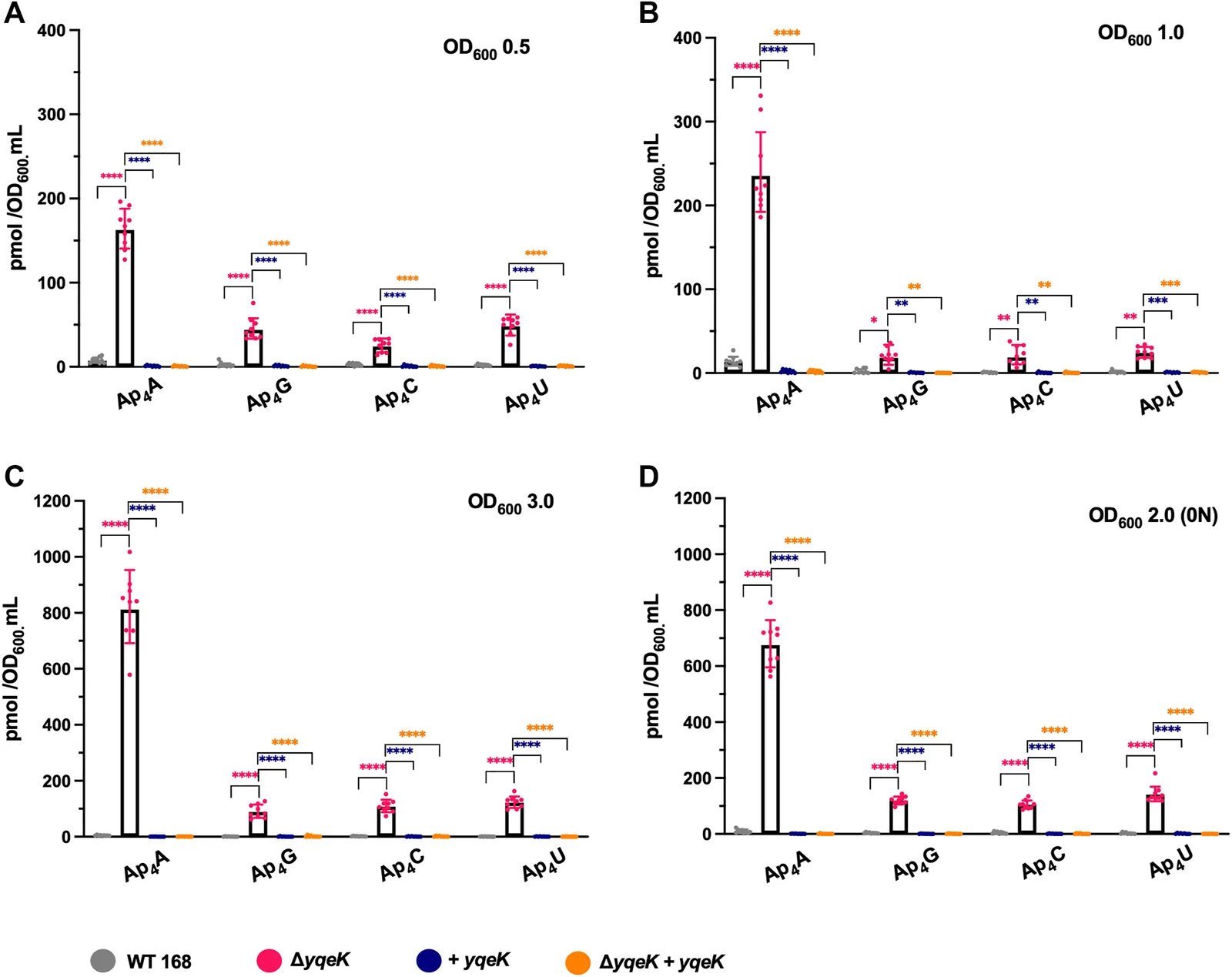
**Quantification of intracellular Ap_4_N dinucleotides.** Targeted QQQ LC–MS/MS analysis of Ap_4_A, Ap_4_G, Ap_4_C and Ap_4_U in *B. subtilis* WT 168, deletion (Δ*yqeK*), overexpression (+*yqeK*) and complementation (Δ*yqeK* + *yqeK*) strains at OD_600_ 0.5 (*A*), 1.0 (*B*), 3.0 (*C*) and overnight culture, OD_600_ 2.0 (*D*). Metabolite levels were normalized to dilution-corrected culture density and are shown as pmol/OD_600_.ml. Bars indicate mean values; points represent nine measurements from three biological replicates, with three technical replicates per clone. Statistical analyses were performed using GraphPad Prism. Technical triplicates were averaged within each biological replicate, and each growth stage was analyzed separately by two-way ANOVA followed by Dunnett’s multiple-comparisons test against Δ*yqeK* within each nucleotide group. Adjusted *p* values are indicated as ∗*p* < 0.05, ∗∗*p* < 0.01, ∗∗∗*p* < 0.001, and ∗∗∗∗*p* < 0.0001.

### Structural basis for promiscuous dinucleoside tetraphosphate recognition by YqeK

To better understand the molecular basis of Ap_4_N degradation and the broad substrate specificity of YqeK, we determined crystal structures of *Gt*YqeK in its apo state and in complex with ADP and the non-hydrolyzable Ap_4_A analog Ap_2_Cp_2_A (Table 2; Fig. 4, *A*–*C*). The overall architecture of YqeK is consistent with that of previously characterized HD-domain phosphohydrolases. Structural comparison with available apo structures of YqeK homologs from other bacterial species revealed a high degree of conservation, with root-mean-square deviations (RMSDs) of 0.7 to 1.0 Å over approximately 160 aligned Cα atoms, indicating that the overall fold is preserved across the family (Fig. S8). The binuclear metal-binding architecture, including the coordinating residues, the relative positions of the metal ions, and overall coordination geometry, is also highly conserved despite differences in the identities of the bound metal ions (Fig. S9). The substrate-binding pocket of *Gt*YqeK harbours two zinc ions that are primarily coordinated by H21, H50, and D127, and by H83, H109, and D51, respectively (Fig. 4*A* and S7*A*). The presence of zinc in the active site was confirmed by X-ray fluorescence (XRF) spectroscopy performed on apo crystals (Fig. S7*B*). We speculate that the zinc ions originated from trace metal contaminants encountered during expression and/or purification of the non-EDTA-treated protein preparation used for crystallization (compare also above). Because the protein was purified in the absence of EDTA, residual Zn^2+^ present in the cells (lysates), buffer components (*e.g.*, phosphate salts, NaCl), chromatography materials, or other laboratory reagents could have been retained by the protein and subsequently visualized in the crystal structure.

**Table 2:** Crystallographic Statistics of Yqek Structures

	Apo-YqeK (PDB-ID: 31LP)	YqeqeK-ADP (PDB-ID: 31LQ)	YqeK-Ap_2_Cp_2_A (PDB-ID: 31LR)
Data collection			
X-ray source	ESRF, Grenoble	DESY, Hamburg	DESY, Hamburg
	Beamline ID30A-3	Beamline P13	Beamline P13
Wavelength (Å)	0.967697	1.05965	0.97625
Space group	*P4*_*1*_*2*_*1*_*2*	*P4*_*1*_*2*_*1*_*2*	*P4*_*1*_*2*_*1*_*2*
Cell dimensions (Å)	73.13 73.13 95.99	73.28 73.28 95.72	73.79 73.79 99.94
Resolution (Å)	45.52–1.65 (1.75–1.65)	47.86–1.70 (1.80–1.70)	46.26–1.40 (1.48–1.40)
Total reflections	872,448 (143,841)	758,538 (125,929)	1,442,830 (217,256)
Unique reflections	31,989 (5087)	29,330 (4635)	54,950 (8734)
Multiplicity	27.27 (28.28)	25.86 (27.17)	26.26 (24.87)
Completeness (%)	100.0 (99.9)	100.0 (100.0)	100.0 (100.0)
*R*_meas_ (%)	11.4 (227.4)	7.5 (161.0)	9.3 (152.9)
*CC*_*1/2*_	99.9 (84.1)	99.9 (88.9)	99.8 (84.0)
*I*/σ(*I*)	19.14 (2.21)	22.44 (1.76)	18.70 (1.67)
Mosaicity (°)	0.044	0.133	0.064
Wilson *B*-factor (Å^2^)	33.87	42.26	31.47
Refinement			
Resolution (Å)	36.57–1.65	34.22–1.70	34.61–1.40
*R*_work_, *R*_free_	0.1784, 0.2002	0.1912, 0.2098	0.1416, 0.1648
Reflections	30,384, 1599	27,830, 1465	52,831, 2099
(working, test set)			
Completeness (%)	99.94	99.88	99.95
r.m.s.d. from ideal			
Bond lengths (Å)	0.006	0.006	0.007
Bond angles (°)	0.742	0.882	0.889
Total number of atoms	1725	1635	1804
Mean *B* value (Å^2^)			
Overall	31.74	45.71	34.28
Protein	30.81	45.50	32.30
Water	40.80	49.92	46.96
Other	35.55	46.73	45.24

Values in parentheses denote the highest resolution shell.

**Figure 4:**
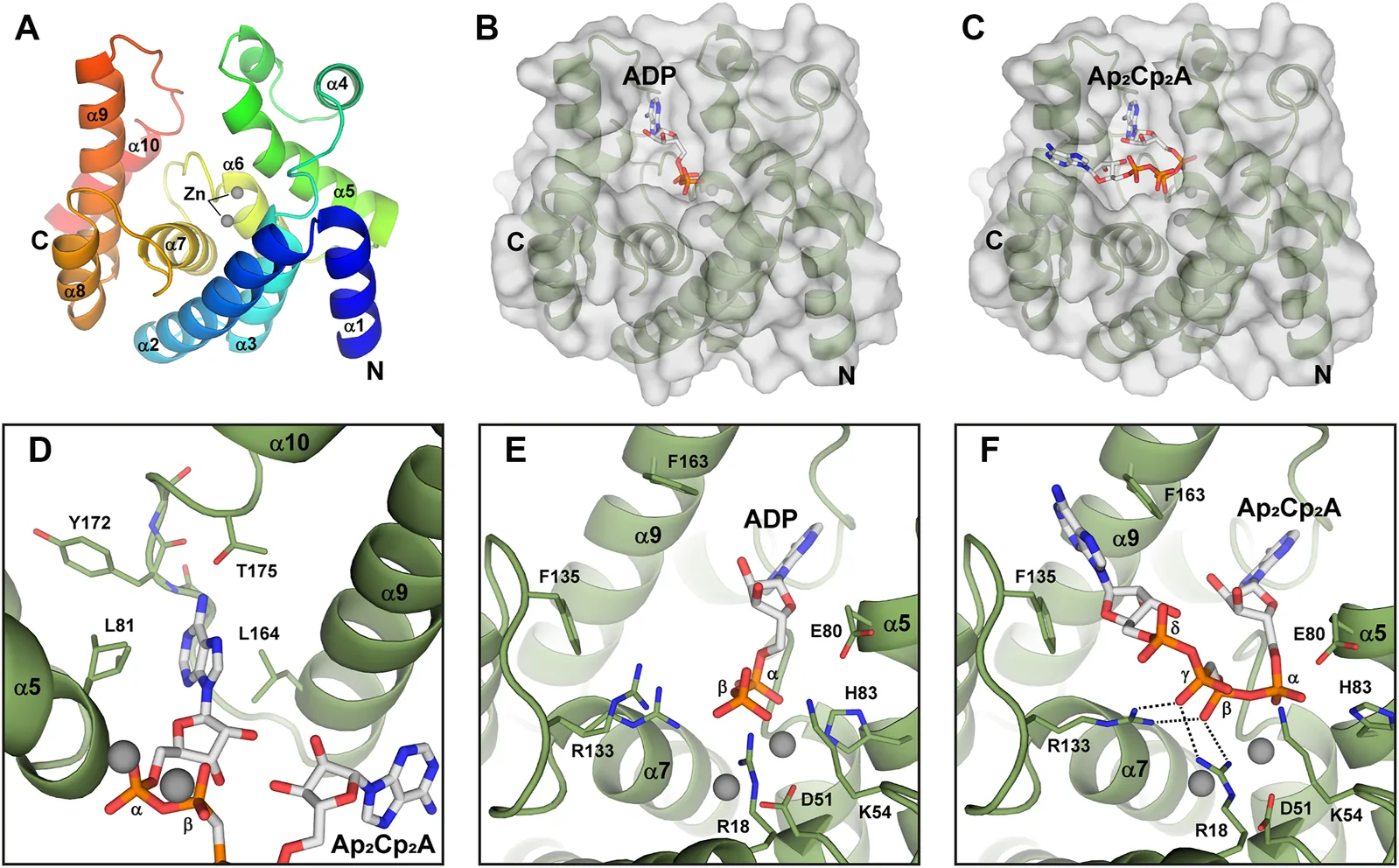
**Structural insights into *Gt*YqeK.** Overall structures of *Gt*YqeK (*A*) in the apo state, (*B*) in a complex with ADP, and (*C*) in a complex with the non-hydrolysable Ap_4_A analog Ap_2_Cp_2_A. (*D*) Detailed view of the deeply buried nucleotide-binding pocket in *Gt*YqeK. *E, F*, structural comparison of the ADP- and Ap_2_Cp_2_A-bound states reveal rearrangements of individual amino acid side chains in the phosphate-binding region and the second nucleotide-binding pocket.

Superposition of the apo and ADP/Ap_2_Cp_2_A-bound structures revealed no major conformational changes upon ligand binding and yielded RMSDs of only 0.142 Å over 178 Cα atoms (apo *versus* ADP) and 0.135 Å over 162 Cα atoms (apo *versus* Ap_2_Cp_2_A), indicating that the nucleotide-binding pocket is largely preformed and does not require substantial structural rearrangements for substrate recognition (Fig. 4, *B* and *C*). The Ap_2_Cp_2_A molecule occupies a surface-exposed binding cleft extending toward the catalytic center (Fig. 4, *C*–*F*). One adenosine moiety is deeply buried within the nucleotide-binding pocket, where the adenine base is stabilized by hydrophobic and hydrogen-bonding interactions. Specifically, the adenine ring engages in π–alkyl interactions with L164 and L81, while additional specificity is provided by hydrogen bonds involving the backbone carbonyl and amide groups of Y172 as well as the hydroxyl group of T175. The same interactions are observed in the *Gt*YqeK-ADP complex. In contrast, the second adenosine moiety of Ap_2_Cp_2_A is positioned closer to the protein surface and is sandwiched between the aromatic side chains of F135 and F163. Binding of the ligand is accompanied by a subtle reorientation of F163 relative to the ADP structure, enabling optimal π–π stacking interactions with the nucleobase (Fig. 4, *D*–*F*).

The tetraphosphate chain is coordinated primarily through electrostatic interactions with a cluster of positively charged residues. The side chains of R18, K54, and R133 contribute the majority of phosphate contacts, whereas H83 and E80 provide additional stabilizing interactions with the shifted α-phosphate. Notably, binding of the phosphate chain induces a reorientation of the guanidinium group of R133, which, together with R18, forms a triangular interaction network around the β- and γ-phosphates of the ligand (Fig. 4*C*). Unexpectedly, all direct protein contacts are restricted to the proximal portion of the dinucleotide. The α-, β-, and γ-phosphates participate extensively in substrate recognition, whereas neither the terminal δ-phosphate nor the ribose moiety of the surface-exposed nucleoside establishes direct contacts with the protein, which resembles the coordination of the outward-facing nucleobase relying predominantly on aromatic stacking. The structure provides a molecular basis for the broad substrate spectrum of YqeK. Whereas one nucleoside moiety and the proximal phosphate groups are recognized through a network of specific interactions that anchor the substrate in the active site, the distal nucleoside is bound predominantly through nonspecific aromatic stacking and remains largely solvent-exposed. This “asymmetric recognition strategy” enables YqeK to tolerate substantial variation in nucleobase identity while maintaining productive substrate binding and catalysis, thereby explaining its promiscuous activity toward diverse Ap_4_N dinucleotides.

### Structure-based mutational analysis of YqeK

To validate our structural analysis and the residues predicted to contribute to Ap_4_A binding and hydrolysis, we performed site-directed mutagenesis. H50 and D51 are located adjacent to the catalytic metal center and were selected to probe the functional importance of the metal-coordination site. R18 and R133 establish key interactions with the polyphosphate chain, whereas F135 and F163 flank the solvent-exposed adenine moiety and are positioned to stabilize substrate binding through aromatic stacking interactions.

The H50A/D51A mutant retained detectable Ap_4_A binding by ITC, although with reduced affinity relative to WT YqeK (*K*_*d*_ = 106 ± 47.6 μM *versus* 74.1 ± 19.6 μM). Despite this residual binding, Ap_4_A hydrolysis was strongly impaired by the H50A/D51A substitution, consistent with a primary role for these residues in metal-dependent catalysis rather than substrate binding alone (Table 3; Figs. S10 and S11). Mutation of the substrate-coordinating residues had a more severe effect. The R18A/R133A mutant, which disrupts the phosphate-binding network, showed no detectable Ap_4_A binding by ITC and no detectable ADP formation by HPLC. Similarly, the F135A/F163A mutant abolished detectable substrate binding and hydrolysis, indicating that aromatic stabilization of the distal nucleobase is required for productive substrate engagement. Together, these mutational data support the structural model in which YqeK activity depends on an intact metal coordination site, arginine-mediated phosphate recognition and aromatic positioning of the nucleobase. This organization enables productive Ap_4_A binding and its metal-dependent hydrolysis. Taken together, these results further strengthen the proposed catalytic mechanism and substrate-binding mode of YqeK.

**Table 3:** Ap_4_A Hydrolysis and Binding Affinity by YqeK Variants

Yqek variants	Relative activity (%)	Binding affinity (*K*_*d*_ in μM)
WT YqeK	100	74.1 ± 19.6
YqeK ^(H50A/D51A)^	No detectable hydrolysis	106 ± 47.6
YqeK ^(R18A/R133A)^	No detectable hydrolysis	No detectable binding
YqeK ^(F135A/F163A)^	No detectable hydrolysis	No detectable binding

Relative Ap_4_A hydrolase activity and ITC-derived Ap_4_A binding affinities of WT YqeK and structural mutants. WT activity was set to 100%. Binding affinities were determined by isothermal titration calorimetry and are reported as *Kd* values in μM. “Not detected” indicates that hydrolysis or binding was below the detection limit under the assay conditions used.

## Discussion

### YqeK coordinates Ap_4_N homeostasis during *B. subtilis* stress adaptation

Our data demonstrate that YqeK is a key regulator of Ap_4_N homeostasis in *B. subtilis* and that its activity is required for optimal fitness under nutrient-limiting conditions. Based on current evidence, YqeK possesses two distinct biochemical activities: it degrades free Ap_4_N dinucleotides (*this study* (29, 30, 31)) and removes Ap_4_N caps from RNA molecules (*this study* (8)). At present, however, it remains unclear whether the physiological phenotypes associated with loss of YqeK arise predominantly from dysregulation of free Ap_4_N pools, altered RNA de-capping, or a combination of both processes.

Notably, deletion of *yqeK* has only minor effects during exponential growth despite causing substantial accumulation of multiple Ap_4_N species. Instead, the most pronounced defects become apparent during prolonged nutrient limitation, including reduced stationary-phase fitness, impaired sporulation, and defective biofilm formation. These observations suggest that the physiological importance of YqeK is linked not simply to the presence of Ap_4_N molecules but rather to their dynamic regulation during adaptation to nutrient-depleted environments. The observation that YqeK overproduction also causes physiological phenotypes further argues that both excessive accumulation and excessive depletion of Ap4Ns are detrimental, implying that precise control of intracellular Ap_4_N levels is required for normal cellular behavior.

Our biochemical and structural analyses provide a mechanistic explanation for how YqeK fulfils this role. Similar to the unrelated bacterial Ap_4_A hydrolase ApaH (5), YqeK catalyzes symmetric cleavage of dinucleoside tetraphosphates and functions as an RNA de-capping enzyme, highlighting striking functional parallels between these two enzyme families. However, YqeK belongs to the HD-domain phosphohydrolases and employs a distinct catalytic architecture. The structure reveals a preformed active site containing a binuclear metal center that coordinates the phosphate chain and promotes metal ion-dependent hydrolysis. Mutational analysis confirms that residues involved in metal coordination are essential for catalysis, whereas residues contacting the phosphate chain and the distal nucleobase are required for productive substrate binding. Together, these data support a model in which YqeK recognizes substrates through a combination of phosphate-chain recognition (*i.e.*, the “p4” of Ap_4_N), anchoring of one nucleoside moiety, and aromatic stabilization of the solvent-exposed distal base. This asymmetric recognition mechanism also explains the remarkable substrate promiscuity of YqeK. Whereas one nucleoside moiety (*i.e.*, the “A” of Ap_4_N) is recognized through specific interactions, the second nucleobase remains largely solvent-exposed and forms predominantly nonspecific aromatic contacts (*i.e.*, “N” of Ap_4_N). This architecture enables efficient hydrolysis of Ap_4_A, Ap_4_G, Ap_4_C, and Ap_4_U, as observed both *in vitro* and *in vivo*, and provides a structural framework for understanding how HD-domain phosphohydrolases accommodate chemically diverse dinucleoside tetraphosphates while maintaining catalytic efficiency.

The broad substrate specificity of YqeK is likely physiologically advantageous. Aminoacyl-tRNA synthetases and other enzymes generate a variety of Ap_4_N species (reviewed in (1, 2)), particularly under nutrient limitation and/or stress conditions, and our metabolomic analyses demonstrate that several of these compounds accumulate in the absence of YqeK. A hydrolase restricted to a single substrate would therefore be insufficient to maintain dinucleotide homeostasis. Instead, YqeK appears to function as a central clearance enzyme that prevents excessive accumulation of multiple signaling dinucleotides.

More broadly, our findings support the emerging view that Ap_4_N molecules are *bona fide* signaling nucleotides rather than merely by-products of aberrant metabolism (*e.g.*, as discussed in: (45)). Together with previous studies demonstrating that Ap_4_A modulates IMPDH activity (6) and regulates protein acetylation through the adaptor AcuB (46, 47), the physiological consequences of altered YqeK activity indicate that Ap_4_N accumulation influences diverse cellular processes. We therefore propose that YqeK serves as a key component of bacterial stress-response networks by controlling the abundance of free and RNA-associated Ap_4_N molecules. Through this function, YqeK enables the transient accumulation of alarmone-like dinucleotides during stress while ensuring their timely removal during adaptation and recovery.

### A binuclear metal center is essential for YqeK function

Our biochemical and structural analyses demonstrate that YqeK is a metal-dependent phosphohydrolase whose catalytic activity relies on a binuclear metal center. Consistent with other members of the HD-domain phosphohydrolase family, coordination of two divalent metal ions is essential for substrate hydrolysis, as substitution of metal-coordinating residues abolished catalytic activity. The crystal structure further shows that the metal ions directly coordinate the phosphate chain of the substrate and position the catalytic water molecule, providing a structural basis for the observed metal dependence.

Although several divalent cations supported YqeK activity *in vitro*, these observations should not necessarily be interpreted as evidence that all of these metals function as physiological cofactors. Metalation of proteins *in vivo* is dictated by intracellular metal availability and homeostasis rather than by the intrinsic catalytic competence of individual metal ions (48). Accordingly, free intracellular Co^2+^ and Ni^2+^ concentrations are maintained at extremely low levels because these metals can be toxic through non-specific protein binding and oxidative stress (*e.g.*, (49, 50)). Instead, they are tightly regulated and selectively delivered to a small number of dedicated metalloenzymes. In contrast, Mn^2+^ is abundant under many physiological conditions and serves as the preferred cofactor for numerous phosphohydrolases. We therefore consider Mn^2+^ to be the most likely physiological metal cofactor for YqeK, whereas activation by Co^2+^ and Ni^2+^ most likely reflects the inherent metal permissiveness commonly observed among HD-domain enzymes. The apparent ability of YqeK to utilize multiple divalent cations *in vitro* may nevertheless have physiological significance. Metal occupancy of metalloenzymes can vary depending on environmental conditions, and fluctuations in intracellular metal availability during nutrient limitation or other stresses could influence YqeK activity. Such modulation would provide an additional regulatory layer linking Ap_4_N turnover to bacterial adaptation. Whether YqeK undergoes metal exchange *in vivo* and whether changes in manganese availability directly affect Ap_4_N homeostasis remain questions for future investigation.

## Experimental procedures

### Strain construction

The *yqeK* gene in *B. subtilis* was disrupted by replacement with a kanamycin resistance cassette, following the strategy described in (51). Approximately 1 kb of DNA fragments upstream and downstream of *yqeK* were amplified by PCR and fused with the kanamycin resistance gene flanked by lox sites using overlap extension PCR. The resulting construct contained the kanamycin cassette between the *yqeK* flanking regions, enabling homologous recombination. The assembled PCR construct was introduced into *B. subtilis* by natural competence transformation, and transformants were selected on LB agar plates containing kanamycin. Correct integration of the kanamycin cassette at the *yqeK* locus was verified by colony PCR using primers annealing outside the flanking regions, and the junctions were confirmed by sequencing. To generate a markerless deletion, the kanamycin cassette was subsequently excised by transient expression of Cre recombinase, resulting in Cre-mediated recombination between the lox sites and leaving a single residual lox site at the locus.

For ectopic gene expression in *B. subtilis*, the integrative plasmid pDR111 was employed. This vector enables stable chromosomal integration at the *amyE* locus through double-crossover homologous recombination and confers spectinomycin resistance for selection of transformants. Gene expression is driven by the IPTG-inducible Phyperspank promoter, allowing controlled and tunable expression of the cloned gene in response to inducer concentration. Genes of interest were amplified by PCR and cloned into pDR111 using standard molecular biology procedures. All plasmid constructs were verified by Sanger sequencing prior to transformation. Recombinant plasmids were introduced into *B. subtilis* strains *via* natural competence, and transformants were selected on LB agar supplemented with spectinomycin. Correct chromosomal integration at the *amyE* locus was verified by colony PCR and phenotypically confirmed by the loss of amylase activity on starch-containing agar plates. The Δ*sigF* mutant strain was generated by natural transformation of the recipient strain with genomic DNA obtained from a previously constructed Δ*sigF* strain kindly provided by Dr Ilka Bischoff (Marburg/Heidelberg). Transformants were selected under the appropriate conditions, and successful transfer of the mutation was confirmed by PCR.

### Sporulation assay

All sporulation assays were performed according to previously established protocols with minor modifications (52). Sporulation was induced by nutrient exhaustion using either DIFCO Sporulation Medium (DSM) or a chemically defined Minimal Medium (SMM), as indicated for individual experiments. Bacterial strains were recovered from glycerol stocks by streaking onto LB agar plates, and single colonies were used to inoculate LB broth. Cultures were incubated overnight at 37 °C with shaking. Overnight cultures were diluted into fresh LB medium and grown to mid-exponential phase to obtain pre-cultures. Cells were subsequently diluted into prewarmed DSM or SMM and incubated at 37 °C with continuous shaking. Sporulation was initiated through nutrient depletion, and cultures were allowed to progress through exponential growth and into stationary phase until maximal sporulation was achieved, typically within 24 to 48 h after inoculation.

Sporulation efficiency and spore formation were monitored by phase-contrast microscopy. For fluorescence-based analyses, culture samples were harvested by centrifugation, washed with phosphate-buffered saline (PBS), and stained with SYTO9 and propidium iodide according to the manufacturer’s instructions (Sigma-Aldrich). Fluorescently stained cells were subsequently analyzed by microscopy or flow cytometry, as appropriate. Where indicated, residual vegetative cells were eliminated by heat treatment at 80 °C for 20 min prior to analysis to enrich for heat-resistant spores.

### Biofilm assay

*B. subtilis* NCIB3610 and 168 derived strains were cultured in LB containing appropriate antibiotics at 37 °C for 14 h. Daily cultures were grown in LB at 37 °C to an OD_600_ of 1.0 without antibiotics. For biofilm growth, bacteria from a liquid LB culture were collected and transferred to solid MSgg medium as previously described [5 mM potassium phosphate (pH 7.0), 100 mM 3-(*N*-morpholino) propane-sulfonic acid (pH 7.0), 2 mM MgCl_2_, 700 μM CaCl_2_, 50 μM MnCl_2_, 100 μM FeCl_3_, 1 μM ZnCl_2_, 2 μM thiamine, 0.5% (v/v) glycerol, 0.5% (w/v) glutamate]. before inoculation (2 μl) on MSgg plates (MSgg medium fortified with 1.5% (w/v) Bacto agar, 6- well plates, dried overnight) and/or 1 mM IPTG (13, 38, 53). Plates were sealed and incubated up to 72 h at 30 °C. For each strain, we analyzed at least 3 biological replicates.

### Spot titer assays

*B. subtilis* strains were recovered from cryo-stocks and streaked onto LB agar plates supplemented with appropriate antibiotics, followed by incubation at 37 °C overnight. A single colony was used to inoculate LB liquid medium, and cultures were grown at 37 °C to mid-exponential phase (OD_600_). Cultures were then normalized to an OD_600_ of 1.0 using fresh LB- or SMM medium. Serial 10-fold dilutions were prepared by transferring 100 μl of culture into 900 μl of sterile phosphate-buffered saline (PBS). Subsequently, 3 μl of each dilution was spotted onto solid LB or SMM agar plates. Plates were incubated at 37 °C for 24 h, followed by an additional 24 h incubation at room temperature.

### Microscopy

Cells cultivated in SM- or DS- medium at 37 °C and 200 rpm were transferred onto 1% (w/v) agarose pads. Images were taken with an Axio Observer.Z1 (Zeiss) microscope equipped with a Plan Apochromat 100×/1.4 Oil Ph3 phase contrast objective and a pco.edge 3.1 sCMOS camera (PCO). An X-Cite 120PC metal halide light source (EXFO) and ET-DAPI, or ET-TexasRed filter cubes GFP missing for SYTO9 (Chroma) were used for fluorescence detection. Images were recorded with VisiView 3.3.0.6 (Visitron Systems, Germany) and processed with Fiji.

### Metabolomics

Bacterial endometabolites were extracted from wild-type and mutant strains by rapid cold methanol quenching followed by methanol/chloroform extraction. Three biological replicates were analyzed for each strain, with three technical replicates prepared from each biological replicate. Strains were streaked from −80 °C glycerol stocks onto LB agar plates and incubated overnight with appropriate antibiotics. Single colonies were used to inoculate LB overnight cultures at 37 °C. Cultures were diluted the following day to OD_600_ = 0.1 in LB medium and grown to exponential phase. These pre-cultures were used to inoculate main cultures in SMM at OD_600_ = 0.1, followed by incubation at 37 °C and 200 rpm. Four sampling points were selected based on growth-curve progression. For quenching, 1 ml of culture was transferred into pre-cooled microcentrifuge tubes containing 1 ml of 70% (v/v) methanol prepared from LC–MS-grade methanol and Milli-Q water. Tubes were pre-cooled at −80 °C for 48 h and handled in a pre-cooled aluminum rack. Samples were mixed by inversion and centrifuged immediately at 13,000×*g* for 10 min at −9 to −10 °C.

Culture density was measured immediately before sampling. High-density cultures were diluted 1:10 with fresh medium before OD_600_ measurement to ensure that the measurements were within the linear range of the spectrophotometer. The measured OD_600_ values were multiplied by the dilution factor of 10. For each sample, 1 ml of culture was harvested, and metabolite amounts were normalized to the dilution-corrected culture density of the harvested sample, expressed as OD_600_ mL. Supernatants were removed, and pellets were stored at −80 °C. For extraction, frozen pellets were kept cold and extracted with OD_600_-normalized volumes of methanol/TE buffer, pH 7.0, 1:1 v/v; TE buffer contained 10 mM Tris and 1 mM EDTA. A volume of 100 μl extraction solvent was used per 1 ml culture at OD_600_ = 0.5 and scaled proportionally. An equal volume of cold chloroform was added, and pellets were resuspended by brief vortexing. Samples were extracted for 2 h with shaking at low temperature, followed by centrifugation at maximum speed for 10 min at −9 to −10 °C. The upper aqueous phase was collected, filtered through a syringe filter and stored at −80 °C until QQQ LC–MS/MS analysis.

### Quantification of nucleotides by HPLC-MS

Nucleotides were quantified using a bio-inert Agilent 1260 Infinity HPLC system coupled to an Agilent 6470 Triple Quadrupole Mass Spectrometer (Agilent Technologies). Chromatographic separation was performed on a SeQuant ZIC-pHILIC column. Solvent A consisted of 10 mM ammonium acetate (pH 9.0) in water, whereas solvent B consisted of 10 mM ammonium acetate (pH 9.0) in 90% (v/v) acetonitrile. A linear gradient was applied from 75% to 40% solvent B over 9 min. Mass spectrometric analysis was carried out using electrospray ionization (ESI) in negative ionization mode. Metabolites were detected by multiple reaction monitoring (MRM), with argon serving as the collision gas. Quantification was performed using external calibration curves covering a concentration range of 0 to 50 μM. Data acquisition and analysis were conducted using MassHunter Workstation Quantitative Analysis for QQQ (Version 10.1, Agilent Technologies).

### Statistical analysis

Statistical analyses were performed using GraphPad Prism. Technical triplicates were averaged within each biological replicate, yielding three independent biological values per condition. Each growth stage was analyzed separately by two-way ANOVA, with nucleotide and strain as factors and including their interaction. Dunnett’s multiple-comparisons test was used to compare WT, *+yqeK*, and *ΔyqeK + yqeK* with the *ΔyqeK* strain within each nucleotide group. Multiplicity-adjusted *p* values below 0.05 were considered statistically significant.

### Protein production and purification

The *yqeK* open reading frames from *B. subtilis* and *G. thermodenitrificans* were amplified from genomic DNA using Phusion High-Fidelity DNA Polymerase and gene-specific primers designed from sequences obtained from NCBI. PCR products were cloned into using the NdeI, XhoI, and BsaI restriction sites. Site-directed mutagenesis was performed using the NEB Q5 Site-Directed Mutagenesis Kit to generate the variants YqeK^(H50A/D51A)^, YqeK^(R18A/R133A)^ and YqeK^(F135A/F163A)^. All constructs were verified by Sanger sequencing and transformed into *E. coli* BL21(DE3) for recombinant protein production. For protein expression, cells were grown in LB medium supplemented with 1% (w/v) D-(+)-lactose monohydrate and incubated at 30 °C for 16 h. Cell pellets were resuspended in lysis buffer (20 mM HEPES, pH 8.0, 250 mM NaCl, 10 mM KCl) and disrupted using an M1-10L Microfluidizer (Microfluidics). Cell debris was removed by centrifugation, and the clarified lysate was subjected to a three-step purification procedure. Hexa-histidine (His_6_)-tagged proteins were initially purified by immobilized metal affinity chromatography (IMAC) using HisTrap HP columns (Cytiva). Columns were equilibrated and washed with lysis buffer supplemented with 40 mM imidazole, and proteins were eluted with 250 mM imidazole. *Bs*YqeK was subsequently treated with 25 mM EDTA to remove residual divalent metal ions and concentrated using Amicon Ultra 10-kDa centrifugal filters (Millipore). The concentrated protein was further purified by anion-exchange chromatography on tandem HiTrap Q HP columns (Cytiva) equilibrated in 20 mM HEPES (pH 8.0) and 50 mM NaCl. Bound proteins were eluted using a linear gradient from 50 mM to 1 M NaCl. Final purification was achieved by size-exclusion chromatography on a HiLoad 16/600 Superdex 200 column (Cytiva) equilibrated in 20 mM HEPES (pH 7.5), 200 mM NaCl, and 20 mM KCl (SEC Buffer). *Gt*YqeK was purified using a two-step procedure comprising nickel-affinity chromatography followed by size-exclusion chromatography under the conditions described above. For metal-dependence assays, *Gt*YqeK was treated with 25 mM EDTA before size-exclusion chromatography. In contrast, the protein used for crystallization was purified without EDTA treatment. Protein purity was monitored by SDS-PAGE following each purification step. Protein concentrations were determined spectrophotometrically using a NanoDrop Lite instrument (Thermo Fisher Scientific). The apparent molecular masses and oligomeric states of purified *Bs*YqeK and *Gt*YqeK were assessed by analytical size-exclusion chromatography using an ÄKTA pure chromatography system equipped with a Superdex 75 Increase 10/300 Gl column (Cytiva). The column was equilibrated with SEC buffer, and protein elution was monitored at 280 nm. Ribonuclease A, ovalbumin, conalbumin, aldolase, and ferritin were used as calibration standards, and apparent molecular masses were estimated from the resulting calibration curve.

### Enzymatic activity assay

Ap_4_A hydrolysis by *Bs*YqeK and *Gt*YqeK was analyzed by HPLC. Endpoint (1 min) reactions were performed in SEC buffer containing 20 mM HEPES (pH 7.5), 200 mM NaCl, and 20 mM KCl, with 1 μM YqeK, 500 μM Ap_4_A, and either 20 or 500 μM of the indicated divalent metal ion (Ca^2+^, Co^2+^, Cu^2+^, Fe^2+^, Mg^2+^, Mn^2+^, Ni^2+^, or Zn^2+^). Assays were performed using EDTA-treated *Bs*YqeK and *Gt*YqeK. *Gt*YqeK purified without EDTA treatment was also analyzed for comparison. For steady-state kinetic analysis, reactions were performed using 5 nM YqeK, 500 μM Mn^2+^ and increasing concentrations of Ap_4_A. Final Ap_4_A concentrations ranged from 1 to 100 μM. All reactions were initiated by addition of enzyme and incubated at 37 °C. Aliquots were removed at 5, 20, 40 and 60 s and quenched with acetonitrile. After centrifugation at 14,000×*g* for 30 min, supernatants were analyzed by HPLC. ADP formation was quantified from integrated peak areas using an ADP standard curve. Initial velocities were calculated from the linear phase of ADP formation over time and fitted to the Michaelis–Menten equation to determine apparent kinetic parameters. HPLC analyses were carried out on an Agilent 1260 Infinity system equipped with a Metrosep A Supp 5-150/4.0 column, using a 10 μl injection volume. Nucleotides were separated isocratically in 90 mM ammonium carbonate (pH 9.25) at a flow rate of 0.6 ml/min, detected by UV absorbance at 260 nm, and identified by comparison with nucleotide standards.

### *In vitro* transcription and decapping of 5′-Ap4A-capped RNA

*In vitro* transcription was performed using a double-stranded DNA template (5′-CAG TAA TAC GAC TCA CTA TT**A** GGC CTC TCG CTCT GCT GGG TGT GCG CTT GC-3′ (54), containing a T7 RNA polymerase promoter sequence and a template sequence (underlined) with a single A at the start to ensure incorporation of Ap_4_A at the 5′ end. The reaction was performed using the HiScribe T7 High Yield RNA Synthesis Kit (New England Biolabs, #E2040S) with the addition of 8 mM Ap_4_A (Jena Bioscience), 10 mM UTP and CTP, and 2 mM GTP. The reaction was incubated for 16 h at 37 °C, followed by DNase I digestion (New England Biolabs, #M0570S) for 15 min and denaturation at 65 °C for 15 min in 2X loading buffer (80% (w/v) formamide, 0.02% (w/v) SDS, 0.02% (w/v) bromophenol blue, 0.01% (w/v) xylene cyanol and 1 mM EDTA). The final transcription product was loaded onto a 15% (v/v) polyacrylamide gel supplemented with 8 M urea, 1X TAE, and 0.2% (w/v) acryloylaminophenyl boronic acid (APB). Ap_4_A-RNA was further gel extracted using the crush and soak method with 3 M sodium acetate (pH 5.3) for 30 min at −80 °C, 5 min at 90 °C, and 16 h at 4 °C with constant mixing. The RNA was subsequently filtered using a 0.45 μm Costar Spin-X Centrifuge Tube Filter (Sigma, #CLS8162-96EA), precipitated with isopropanol at −80 °C for 16 h, washed twice with 70% (v/v) ethanol and resuspended in nuclease-free water. To determine decapping activity, 2 μM of *Bs*YqeK protein (or *Ec*ApaH) was incubated with 100 ng of 5′-Ap_4_A-capped RNA in a reaction buffer containing 50 mM Tris-HCl (pH 8.0), 200 mM NaCl and 10 mM of MgCl_2_ for 30 min at 37 °C in a 10 μl reaction volume. The reaction was then denatured in 2X loading buffer at 65 °C for 15 min and run on a 15% (v/v) polyacrylamide gel supplemented with 8 M urea, 1X TAE and 0.2% (w/v) APB. The final decapping products were visualised with the InstaS GelStick Imager (InstaS Science Imaging) after staining with SYBR Gold Nucleic Acid Gel Stain solution (ThermoFisher, #S11494).

### Isothermal titration calorimetry

Binding of nucleotide substrates to *Bs*YqeK was measured by isothermal titration calorimetry. Purified WT YqeK and YqeK variants were buffer-exchanged into SEC buffer before analysis, and nucleotide ligands were prepared in the same buffer to minimize heat of dilution. Protein and ligand solutions were clarified by centrifugation before loading. All ITC experiments were performed with 50 μM YqeK in the cell at 25 °C. Nucleotide substrates were loaded into the syringe and titrated into protein. WT YqeK was titrated with Ap_4_A at 2.5 mM, Ap_4_G at 1.5 mM, Ap_4_C at 2 mM and Ap_4_U at 2 mM. YqeK variants were analyzed using Ap_4_A as the ligand under the same protein concentration, with Ap_4_A loaded in the syringe. Raw injection heats were integrated and fitted using a single-site binding model. Dissociation constants (*K*_*d*_) were obtained from the fitted binding isotherms. Experiments with no saturable heat change above background were reported as showing no detectable binding under the assay conditions used.

### Crystallization and structure determination

Initial crystallization attempts with YqeK from *B. subtilis* did not yield diffraction-quality crystals. Therefore, the homolog from *G. thermodenitrificans* was selected, as proteins from thermophilic organisms are typically more stable and often crystallize more readily than those from mesophilic species. *Gt*YqeK purified without EDTA treatment was used for all crystallization experiments. Crystals of apo *Gt*YqeK, *Gt*YqeK in complex with ADP, and *Gt*YqeK in complex with a non-hydrolysable Ap_4_A analog (methyl-bridged between the four phosphate moieties) (Biolog) were obtained by the sitting-drop vapor diffusion method at 20 °C. Drops consisting of equal volumes of protein solution (1.5 mM) and reservoir solution were dispensed with a Gryphon robotic system into SWISSCI MRC 3-well plates. For complex formation, ADP or Ap_2_Cp_2_A was added to the protein solution at a final concentration of 20 mM and 10 mM, respectively. Initial screening was carried out with the NeXtal JCSG Core Suites I-IV. Apo crystals of *Gt*YqeK were obtained from crystallization conditions containing 0.1 M sodium/potassium phosphate and 2.5 M sodium chloride. Crystals of *Gt*YqeK in complex with ADP were obtained from conditions comprising 0.05 M calcium acetate, pH 8.0, 0.1 M imidazole, and 35% (v/v) 2-ethoxyethanol as the precipitant. Crystals of *Gt*YqeK in complex with Ap_2_Cp_2_A were obtained from conditions containing 0.4 M potassium/sodium tartrate. Prior to data collection, all crystals were cryoprotected with 30% (v/v) glycerol and flash-frozen in liquid nitrogen. X-ray diffraction data were collected at 100 K at beamline ID30A-3 (ESRF) for apo *Gt*YqeK and at beamline P13 (DESY) for *Gt*YqeK in complex with ADP and the non-hydrolyzable Ap_4_A analog Ap_2_Cp_2_A. To identify bound metals, X-ray fluorescence (XRF) scans of apo crystals were performed at beamline ID30B (ESRF). Diffraction data were processed with XDS (55) using XDSAPP3 (https://doi.org/10.1107/S0021889812011715). Initial phases were obtained by molecular replacement using the phenix.phaser module (56) of the PHENIX suite (57) with the AlphaFold3 model (58) of *Gt*Yqek as search model (TFZ = 45.0; LLG = 1538). The refined apo structure was subsequently used to solve the *Gt*YqeK-ADP (TFZ = 81.4; LLG = 6983) and *Gt*YqeK-Ap_2_Cp_2_A (TFZ = 79.3; LLG = 13,208) complex structures. After automated model building with phenix.autobuild (59), final models were obtained through iterative cycles of manual model building in COOT (60, 61) and refinement with phenix.refine (62). Ligand restraints for Ap_2_Cp_2_A were generated with Grade2 (Global Phasing Ltd; https://grade.globalphasing.org/).

## Data availability

Coordinates and structure factors have been deposited in the Protein Data Bank under accession codes 31LP (apo *Gt*YqeK), 31LQ (*Gt*YqeK-ADP) and 31LR (*Gt*YqeK-Ap_2_Cp_2_A), respectively.

## Supporting information

This article contains supporting information.

## Conflict of interest

The authors declare that they have no conflicts of interest with the contents of this article.
