# Supplementary material for: Molecular insights into the promiscuous Ap4N hydrolase YqeK
Source: J Biol Chem. 2026 Aug 25;302(10):113490. doi: 10.1016/j.jbc.2026.113490 (PMC13627108; doi:10.1016/j.jbc.2026.113490)
Supplement: Supplementary file 1 [file mmc1.pdf]

Supplementary Information for:

Molecular insights into the promiscuous Ap_4N hydrolase YqeK

Renuka Dharani Shivakumar[1,2], Natalie Happel[1,2], Fabiana Burchert[1,2], Michel Brück[3], Lennart Randau[1,3,5], Jan Pané-Farré[1,2], Uwe Linne[2], Johannes Freitag[1,3], Rebecca Hinrichs[1,6], Stephan Kiontke[1,2], Gert Bange[1,2,4,5,7], Patricia Bedrunka-Meinert[1,2,7]

[1]Center for Synthetic Microbiology (SYNMIKRO), Philipps University Marburg
[2]Department of Chemistry, Philipps University Marburg
[3]Department of Biology, Philipps University Marburg
[4]Max Planck Institute for terrestrial Microbiology, Marburg
[5]Microbes-for-Climate (M4C) Cluster of Excellence, Marburg, Germany.
[6]Microcosm Earth Center (MEC), Max Planck Institute for Terrestrial Microbiology and Philipps University, Marburg
[7]To whom correspondence should be addressed: gert.bange@synmikro.uni-marburg.de, patricia.bedrunka@synmikro.uni-marburg.de

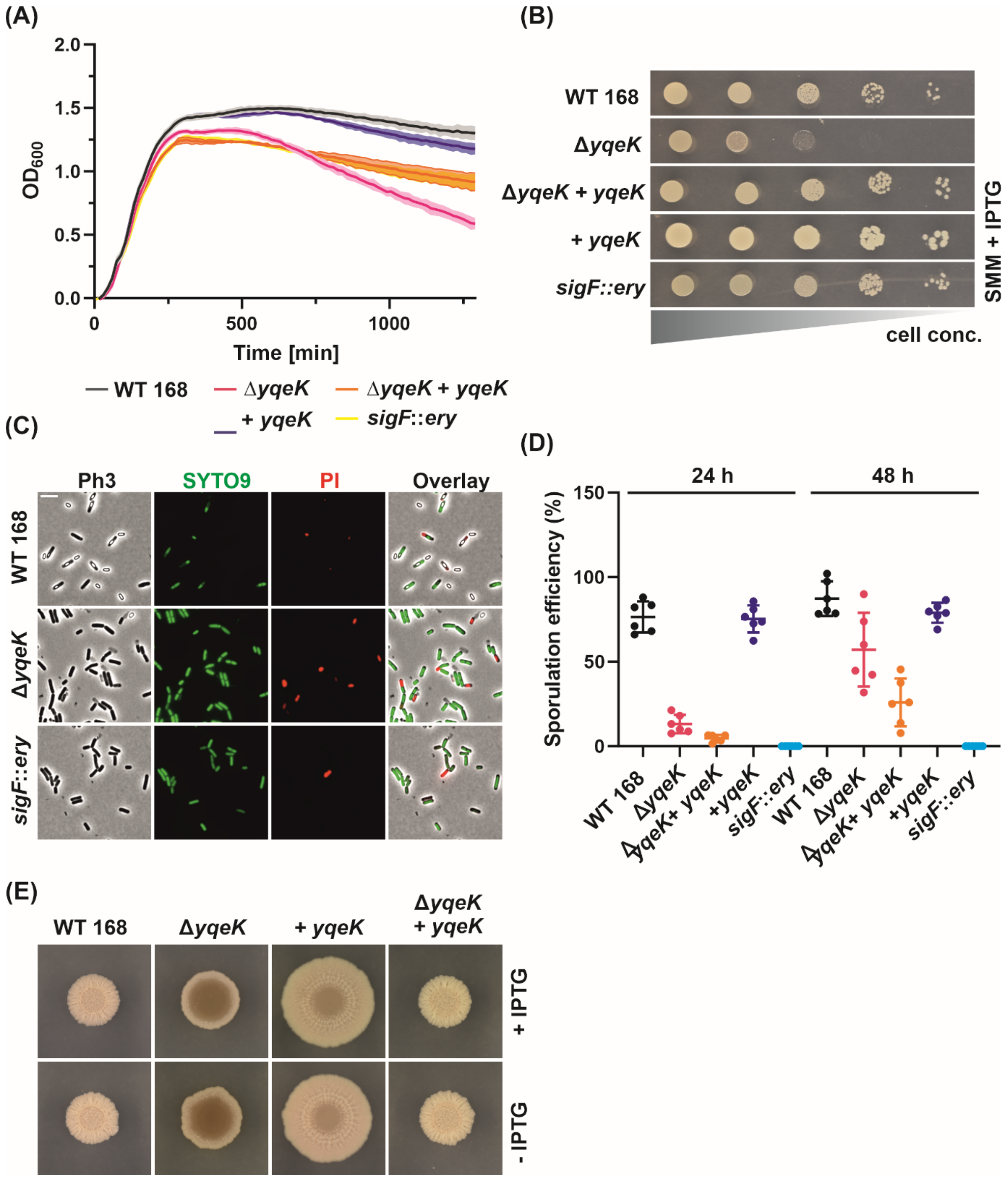

Fig. S1. Additional phenotypic consequences of *yqeK* deletion in *B. subtilis* in different WT backgrounds. (A) Growth of the indicated strains in LB medium at 37 °C, monitored by OD_{600} using a plate reader. Lines show mean values and shaded areas indicate standard deviation (s.d.) from three technical replicates. **(B)** Spot-titer assay of exponentially growing cultures on SMM agar supplemented with 1 mM IPTG. Serial dilutions are shown from left to right. **(C)** Phase-contrast and fluorescence microscopy of the indicated strains after 24 h growth in SMM medium. Cells were stained with SYTO9 and propidium iodide (PI). Scale bar, 5 µm. **(D)** Sporulation efficiency of the indicated strains after 24 h and 48 h. Points indicate individual measurements; bars show mean ± s.d. **(E)** Colony morphology of WT 168, Δ*yqeK*, +*yqeK* and Δ*yqeK* + *yqeK* strains grown on SMM agar in the presence or absence of IPTG.

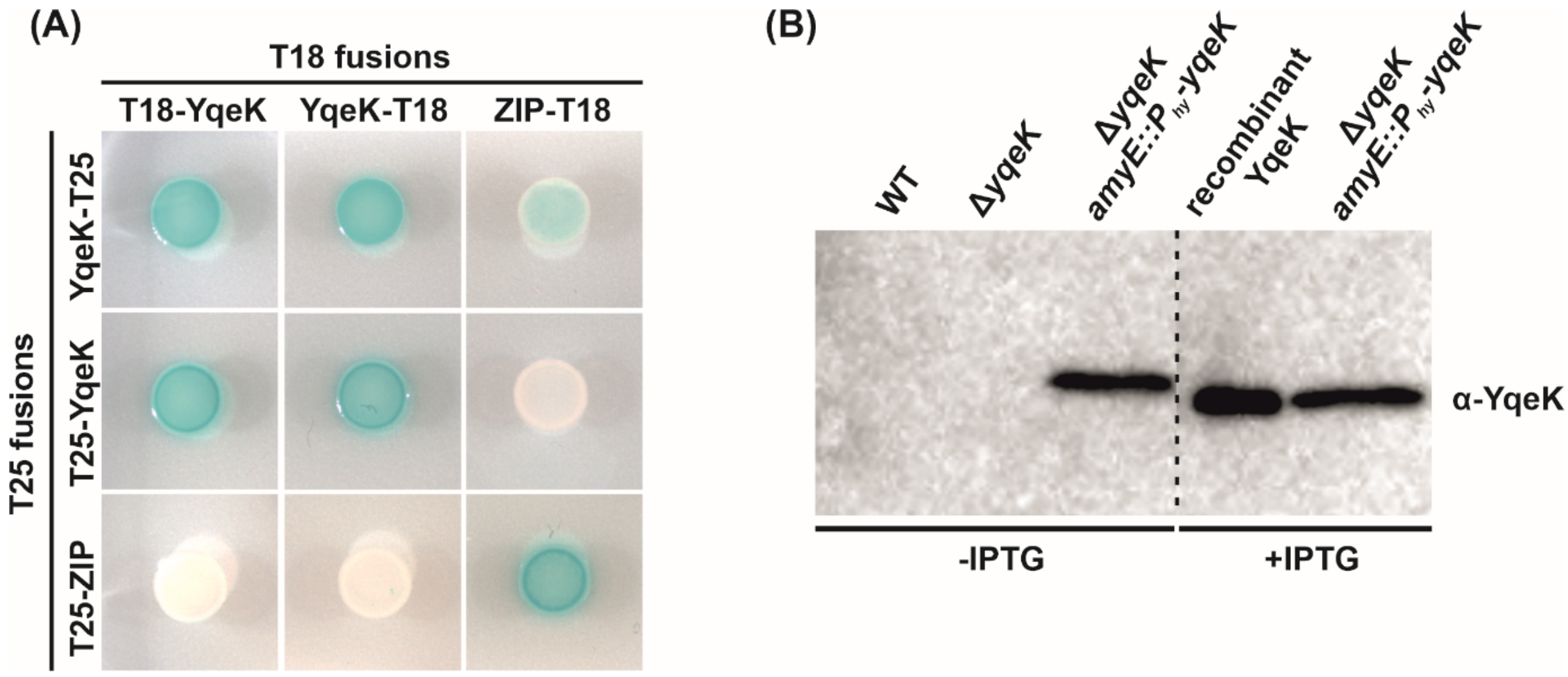

Fig. S2. YqeK dimerization and expression. (A) Bacterial two-hybrid analysis of YqeK self-interaction using reciprocal T18 and T25 fusion constructs. Blue colonies indicate interaction-dependent reporter activation. ZIP fusions served as positive and negative controls. **(B)** Immunoblot detection of YqeK in WT, Δ*yqeK*, complemented and recombinant expression strains grown in LB to exponential phase with or without IPTG. YqeK was detected using an anti-YqeK antibody.

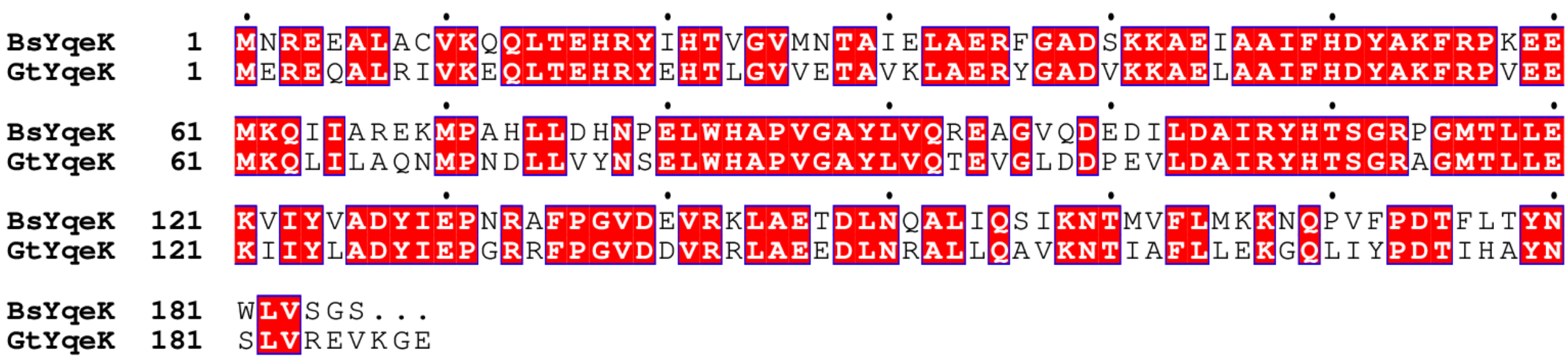

Fig. S3. Sequence alignment of YqeK from *B. subtilis* and *G. thermodenitrificans*. Identical residues are highlighted in red with white lettering.

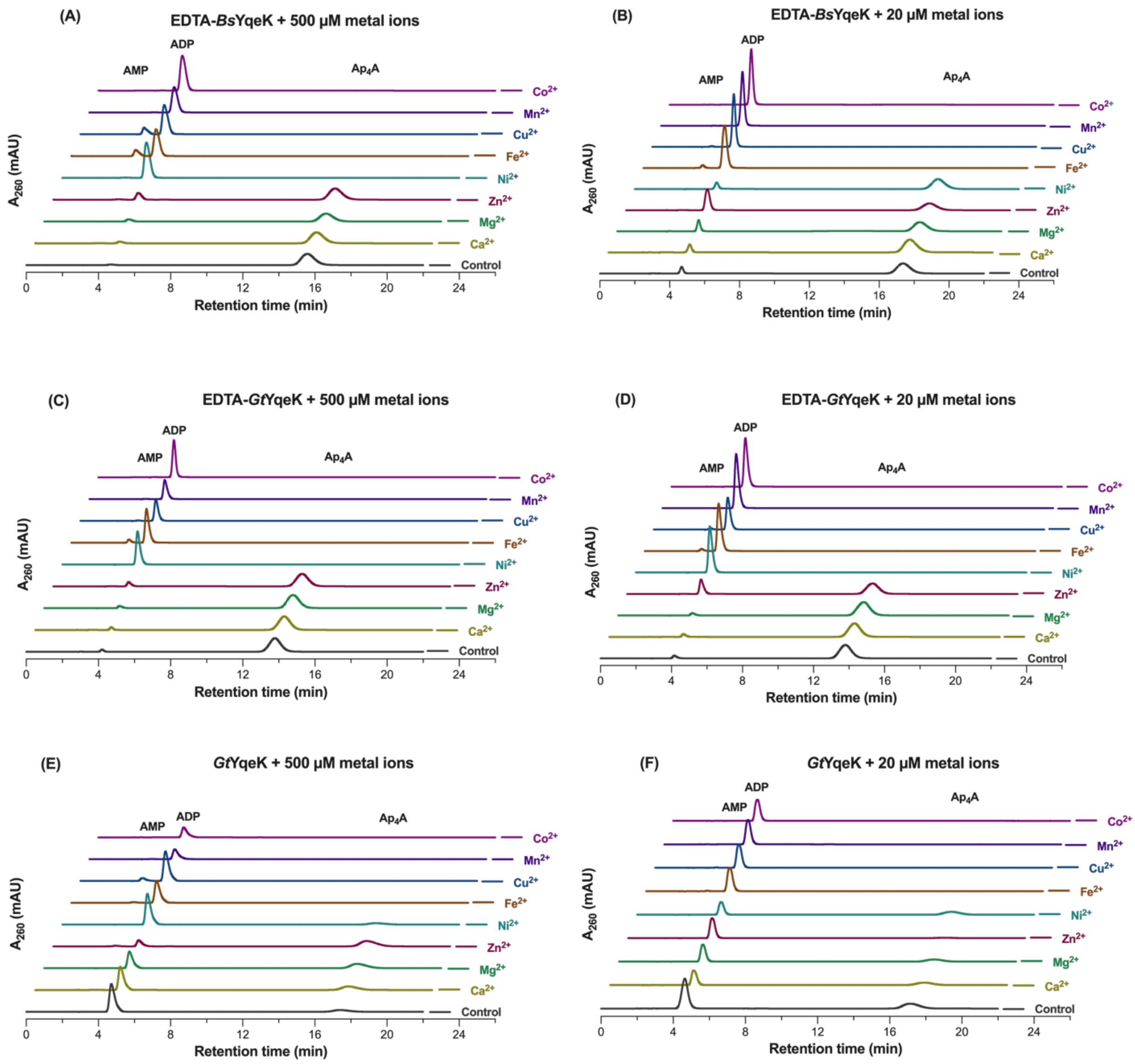

Fig. S4. Effects of divalent metal ions on Ap_4A hydrolysis by *Bs*YqeK and *Gt*YqeK. HPLC analysis of Ap_4A hydrolysis by EDTA-treated *Bs*YqeK (A, B), EDTA-treated *Gt*YqeK (C, D), and *Gt*YqeK purified without EDTA treatment (E, F) in the presence of 500 µM (A, C, E) or 20 µM (B, D, F) of the indicated divalent metal ions. Reactions containing 1 µM YqeK and 500 µM Ap_4A were incubated at 37 °C for 1 min. Nucleotides were detected by UV absorbance at 260 nm and identified by comparison with authentic standards analysed under the corresponding chromatographic conditions. Chromatograms are vertically offset for clarity, and the retention positions of AMP, ADP, and Ap_4A are indicated. Differences in Ap_4A retention time between panels reflect run-to-run chromatographic variation.

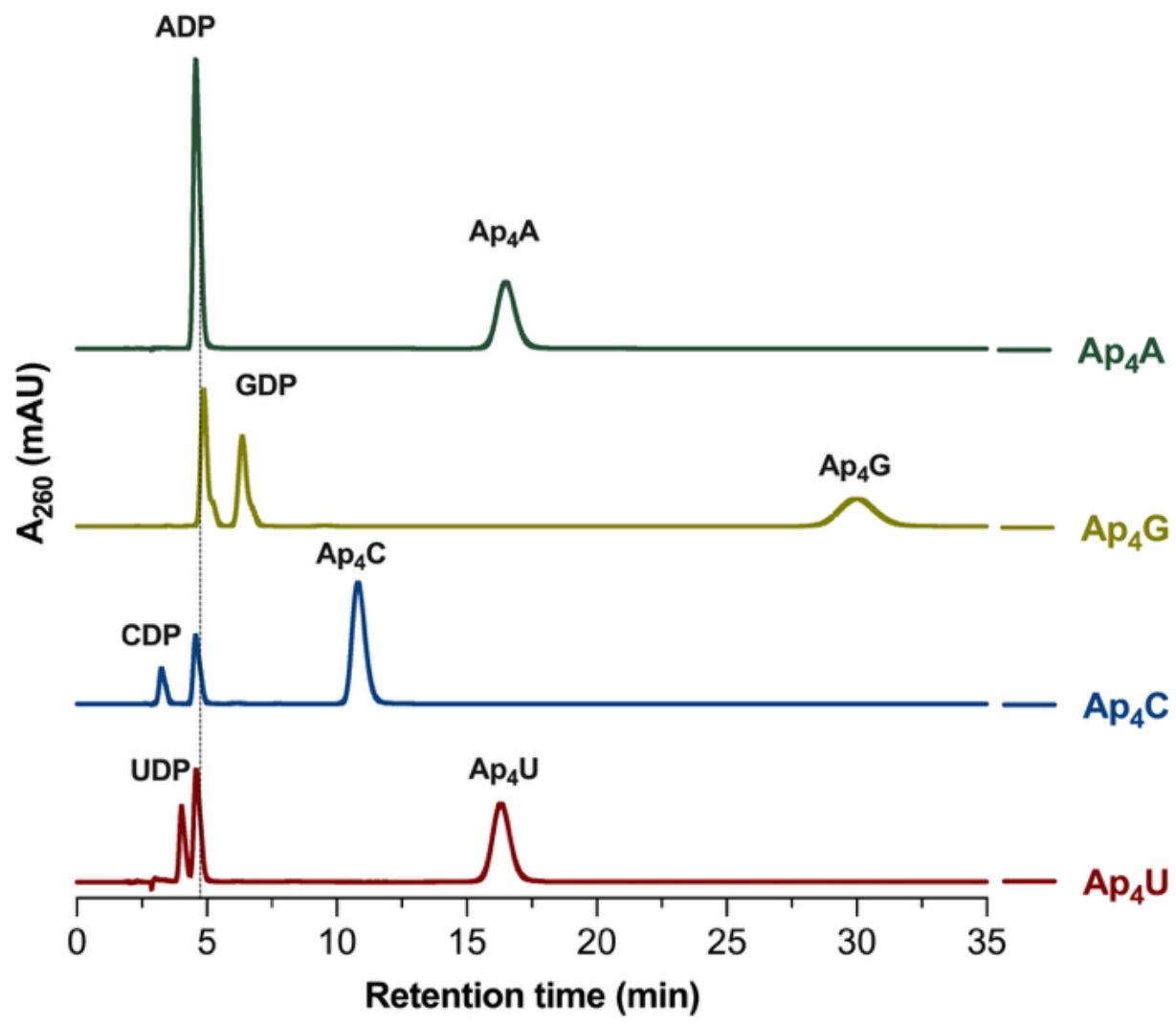

Fig. S5: Substrate promiscuity of *Bs*YqeK. HPLC analysis of *Bs*YqeK activity towards Ap_4N dinucleotide substrates in the presence of manganese. Reaction mixtures containing 5 nM enzyme, 6 µM substrate and 500 µM Mn^{2+} were incubated for 1 min at 37 °C and analysed under identical chromatographic conditions. *Bs*YqeK hydrolyzed Ap_4A and mixed-base Ap_4N substrates, including Ap_4C, Ap_4G and Ap_4U, with ADP detected as the main product across the substrate panel. Additional substrate-specific products, including CDP, GDP and UDP, are indicated where detected. Products and substrates were assigned by comparison with nucleotide standards using the following retention times: ADP, 4.9 min (indicated as dashed line); Ap_4A, 16.5 min; CDP, 3.8 min; Ap_4C, 12.2 min; GDP, 6.3 min; Ap_4G, 29.0 min; UDP, 4.2 min; Ap_4U, 16.7 min.

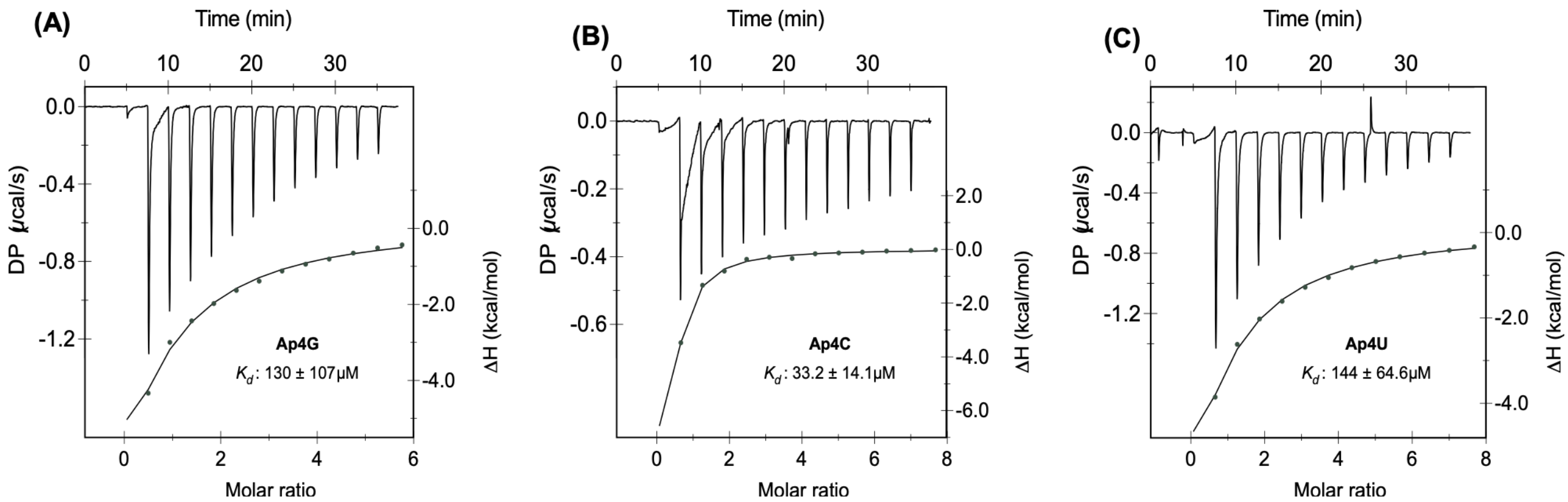

Fig. S6. Binding of *Bs*YqeK to mixed Ap_4N dinucleotides. ITC measurements showing the interaction of *Bs*YqeK with the mixed-base dinucleotides Ap_4G **(A)**, Ap_4C **(B)** and Ap_4U **(C)**. Each panel shows the raw thermogram above and the corresponding integrated binding isotherm below. The fitted dissociation constants were K_d = 130 ± 107 µM for Ap_4G, 33.2 ± 14.1 µM for Ap_4C and 144 ± 64.6 µM for Ap_4U, indicating micromolar binding across the tested Ap_4N substrates.

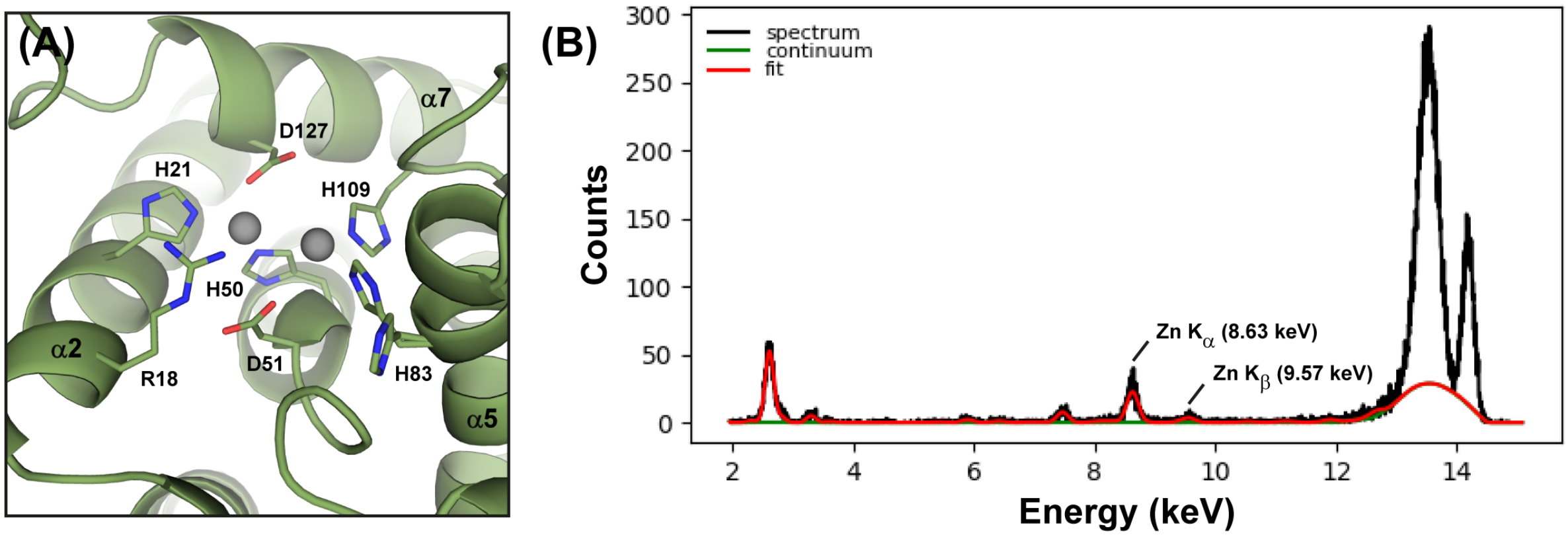

Fig. S7. The metal-binding pocket of *Gt*YqeK harbors Zn^{2+} ions. (A) Structural overview of Zn^{2+} coordination within the nucleotide-binding pocket of *Gt*YqeK. (**B**) X-ray fluorescence (XRF) spectrum recorded over an energy range of 2-15 keV (incident X-ray energy: 14.2 keV), confirming the presence of Zn^{2+} ions by the characteristic Zn K_a and K_b emission peaks.

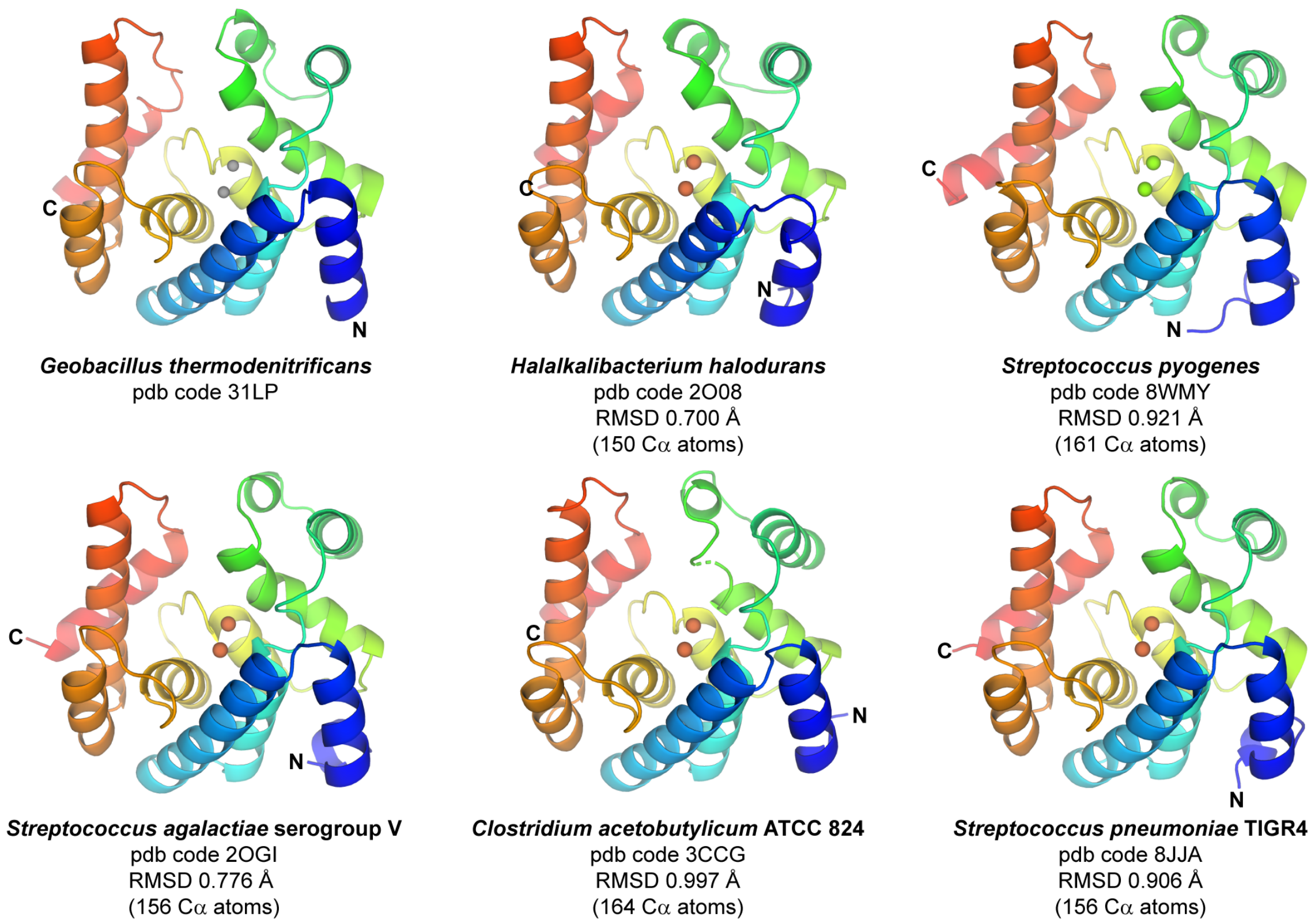

Fig. S8. Structural comparison of YqeK homologs. Ribbon representations of YqeK homologs from *G. thermodenitrificans* (this study) and structurally related proteins from *Halalkalibacterium halodurans* (PDB: 2O08), *Streptococcus pyogenes* (PDB: 8WMY), *Streptococcus agalactiae* serogroup V (PDB: 2OGI), *Clostridium acetobutylicum ATCC 824* (PDB: 3CCG), and *Streptococcus pneumoniae* TIGR4 (PDB: 8JJA). Structures are shown in the same orientation and colored from the N-terminus (blue) to the C-terminus (red). Conserved metal ions located within the central cavity are depicted as spheres: Zn (gray), Fe (dark red), and Mg (green). Root-mean-square deviation (RMSD) values and the number of aligned Cα

atoms are indicated for each structural superposition, highlighting the high degree of structural conservation across YqeK-family proteins.

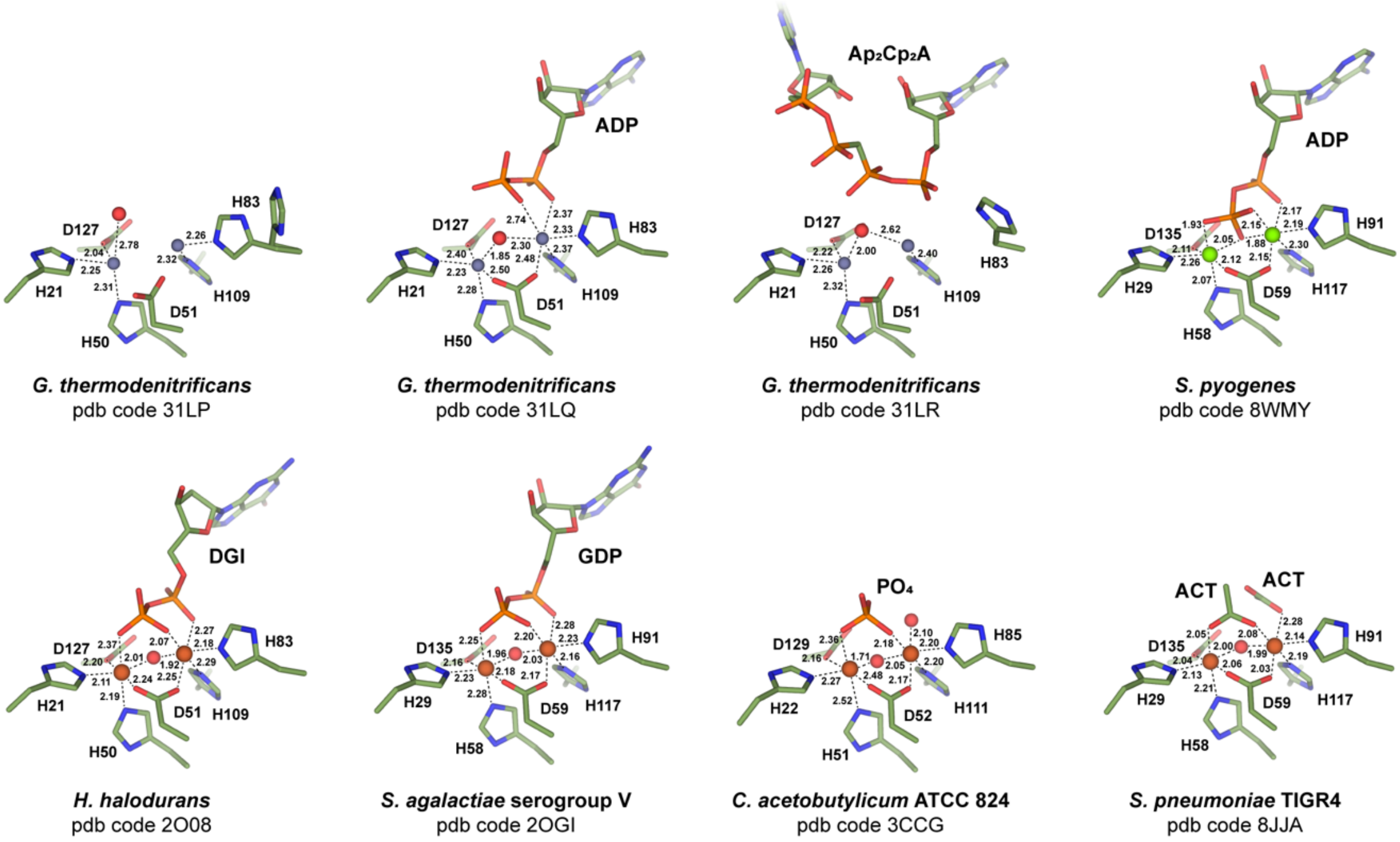

Fig S9. Conserved architecture of the YqeK binuclear metal-binding site. Comparison of the active sites of *G. thermodenitrificans* YqeK (PDBs: 31LP, 31LQ, and 31LR) with representative homologous YqeK structures from *S. pyogenes* (PDB: 8WMY), *H. halodurans* (PDB: 2O08), *S. agalactiae* serogroup V (PDB: 2OGI), *C. acetobutylicum* ATCC 824 (PDB: 3CCG), and *S. pneumoniae* TIGR4 (PDB 8JJA). Metal-coordinating residues and ligands are shown as sticks, metal ions as colored spheres, and water molecules as red spheres. Dashed lines indicate selected coordination interactions and interatomic distances (Å).

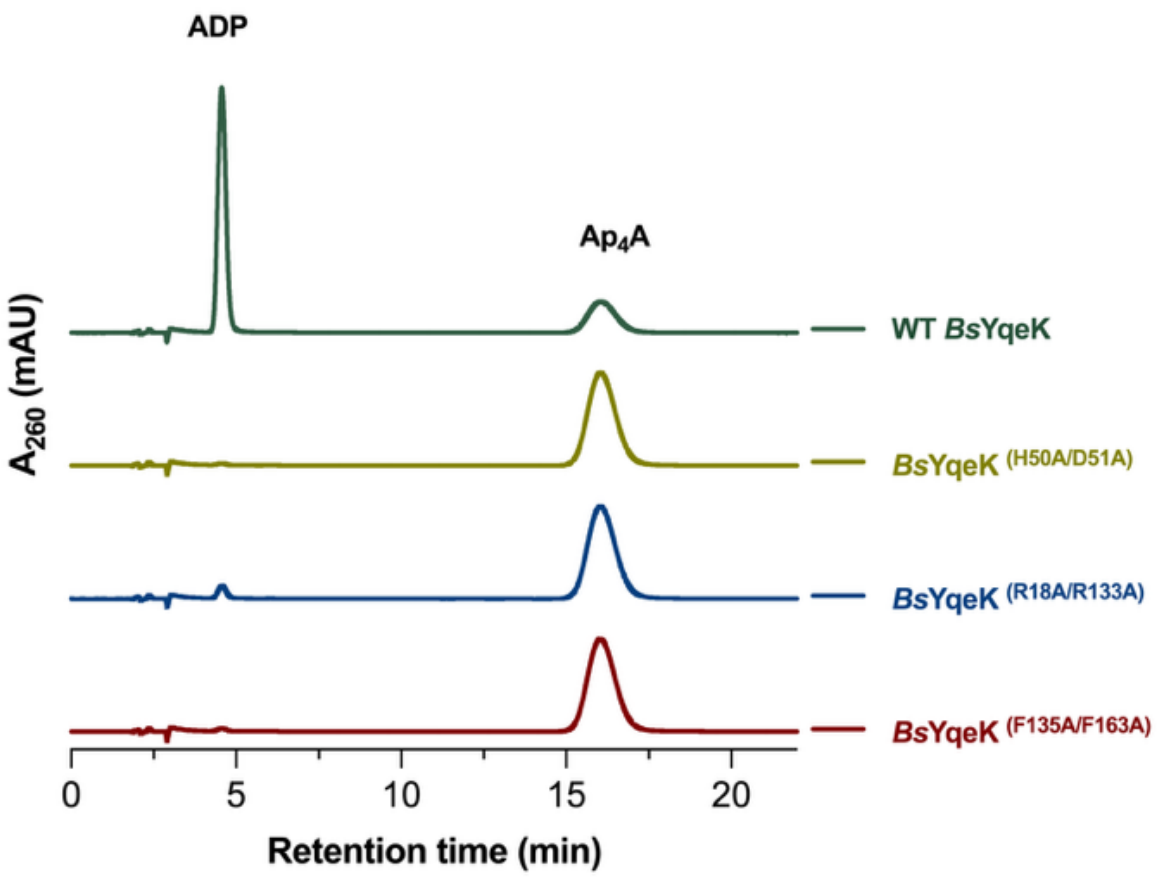

Fig. S10. Ap₄A-degrading activity of different *Bs*YqeK variants. Ap₄A hydrolysis by wild-type (WT) *Bs*YqeK and the indicated *Bs*YqeK variants was monitored by HPLC with UV detection at 260 nm. Reaction mixtures containing 5 nM enzyme and 6 µM Ap₄A with 500 µM Mn^{2+} were incubated for 1 minute at 37 °C and analyzed under identical chromatographic conditions. WT *Bs*YqeK generated a prominent ADP peak (retention time - 4.5 min) accompanied by a reduction in the Ap₄A peak (retention time ~16 min), whereas the H50A/D51A, R18A/R133A, and F135A/F163A variants displayed substantially diminished product formation and retained higher levels of Ap₄A. Peak identities were assigned by comparison with authentic nucleotide standards.

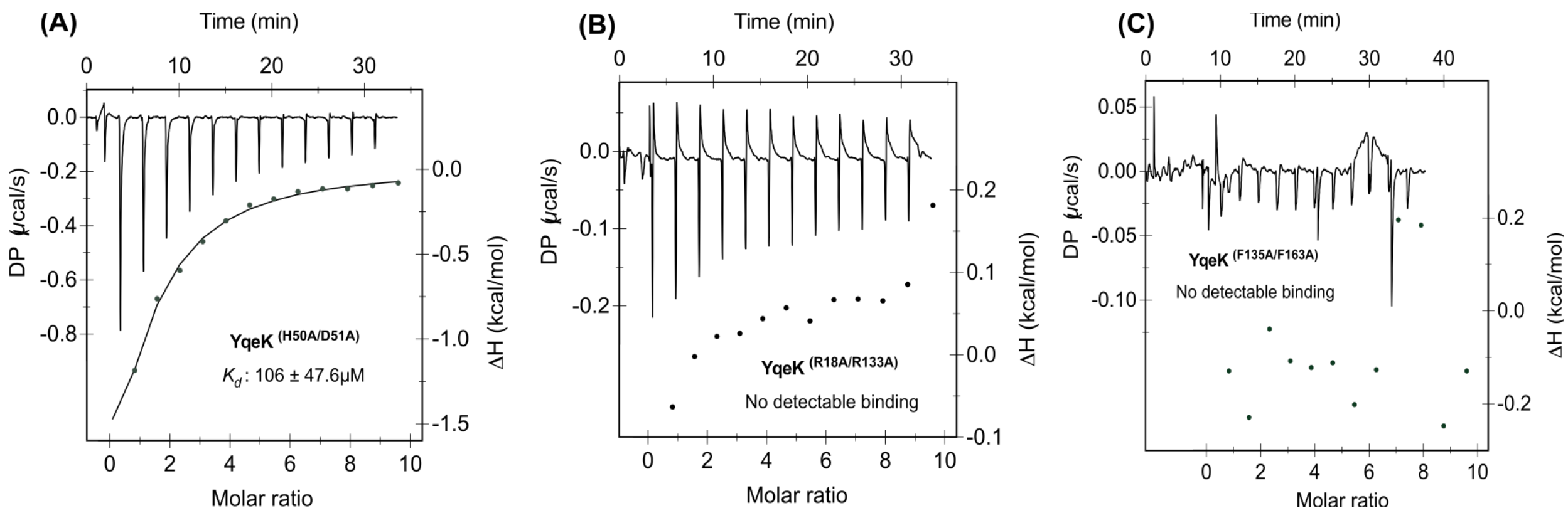

Fig. S11. Ap₄A binding by *Bs*YqeK active-site variants. ITC analysis of Ap₄A binding to *Bs*YqeK active-site variants. **(A)** *Bs*YqeK $^{(H50A/D51A)}$ retained Ap₄A binding, with a fitted K_d of 106 ± 47.6 µM. **(B)** *Bs*YqeK $^{(R18A/R133A)}$ and **(C)** *Bs*YqeK $^{(F135A/F163A)}$ showed no detectable Ap₄A binding. Raw injection heats are shown in the upper panels, with integrated heats and binding fits shown below.

Table S1. Strains used in this study

Strain	Relevant genotype	Source
B. subtilis 168	wild type, *trpC2*	BGSC, 3A38
B. subtilis NCIB3610	wild type, prototroph, $comI^{Q12L}$	(1)
E. coli DH5α	*recA1 endA1 lacZΔM15*	Lab. stock
E. coli BL21(DE3)	DE3 lysogen; T7 RNA polymerase expression strain	Lab. stock
G. thermodenitrificans NG-80	wild type thermophilic bacterium	Lab. stock
**Derivatives of *B. subtilis* 168**		
RS22	*yqeK*::*kan (kan)*	This study
RS24	Δ*yqeK*	This study
RS34	*amy*::P_{IPTG}-*yqeK* (*spec*)	This study
RS38	Δ*yqeK*, *amy*::P_{IPTG}-*yqeK* (*spec*)	This study
NH52	*sigF*::*ery (ery, lin)*	This study
**Derivatives of *B. subtilis* 3610**		
RS23	*yqeK*::*kan (kan)*	This study
RS25	Δ*yqeK*	This study
RS35	*amy*::P_{IPTG}-*yqeK* (*spec*)	This study
RS39	Δ*yqeK*, *amy*::P_{IPTG}-*yqeK* (*spec*)	This study
NH78	*sigF*::*ery (ery, lin)*	This study

Table S2. Plasmids used in this study

Plasmid	Relevant features	Insert/mutations	Source
**pET24d**	T7/lac expression; (*kan)*		Lab. stock
**pRS031**	pET24d - C`His_6-*Bs*YqeK; T7/lac expression; (*kan)*	*Bs*YqeK	This study
**pRS042**	pET24d - C`His_6-*Gt*YqeK; T7/lac expression; (*kan)*	*Gt*YqeK	This study
**pRS077**	pET24d - C`His_6-*Bs*YqeK$^{(H50A/D51A)}$; T7/lac expression; (*kan)*	*Bs*YqeK$^{(H50A/D51A)}$	This study
**pRS105**	pET24d - C`His_6-*Bs*YqeK$^{(R18A/R133A)}$; T7/lac expression; (*kan)*	*Bs*YqeK$^{(R18A/R133A)}$	This study
**pRS106**	pET24d - C`His_6-*Bs*YqeK$^{(F135A/F163A)}$; T7/lac expression; (*kan)*	*Bs*YqeK$^{(F135A/F163A)}$	This study
**pDR111**	*amyE*::Phy, spcR ectopic integration vector with a strong IPTG inducible promoter		kind gift of D. Rudner (Boston, MA, US)
**pRS084**	*amy*::P_{IPTG}-*yqeK*; (*spec*)	*Bs*YqeK	This study
**pDR244**	Temperature-sensitive Cre-expression plasmid used for excision of lox-flanked kan resistance cassettes.		Lab. stock

Table S3. Primers used in this study

Primer name	Sequence, 5'–3'	Purpose	Construct
**RS031F**	ttaaggtctcccatgggcaatcgtgaggaggcacttgcatgcgtg	Amplification of *Bs*Yqek	pRS031
**RS031R**	ttaaggtctcctcgagttagtgatggtgatggtgatgtcagctgccagacacaagccag		
**RS042F**	ttaaggtctcccatgggcgagcgagaacaagcgttgcgg	Amplification of *Gt*Yqek	pRS042
**RS042R**	ttaaggtctcctcgagttagtgatggtgatggtgatgttcccccttcacttcg		
**RS076F**	ggcaatctttgctgattacgccaaattc	Site directed mutagenesis of *Bs*Yqek H50A	pRS077
**RS076R**	gctatttccgcttttttag		
**RS077F**	atctttgctgcttacgccaaattcc	Site directed mutagenesis of *Bs*Yqek D51A	
**RS077R**	tgccgctatttccgcttt		
**RS101F**	gaccgaacacgcatatatccatacgg	Site directed mutagenesis of *Bs*Yqek R18A	pRS105
**RS101R**	aattgctgcttcacg		
**RS102F**	tgagccaaacgcggcatttcccg	Site directed mutagenesis of *Bs*Yqek R133A	
**RS102R**	atgtaatcggcaacg		
**RS103F**	aaaccgggcagctcccggtgtcg	Site directed mutagenesis of *Bs*Yqek F135A	pRS106
**RS103R**	ggctcaatgtaatcggcaac		
**RS104F**	tacaatggttgctcttatgaaaaaaaatcaac	Site directed mutagenesis of *Bs*Yqek F163A	
**RS104R**	tttttaatggactgaatcagc		
**RS084F**	ttaagtcgacgtatcatcaactatgataagggaac	Amplification of *Bs*Yqek	pRS084
**RS051R**	ttaagctagctcagctgccagacacaagc		
**RS082_5pL**	caacgtctgtgggcatgtatc	Amplification of *Bs*Yqek Upstream	RS082_Up
**RS082_5pR**	ctctcctttctcgcctgcctgcttcacgcatgcaagtg		
**RS082_3pL**	gcagtgacaggagcctcgagctgaacaggaggaatttg	Amplification of *Bs*Yqek Downstream	RS082_Dw
**RS082_3pR**	gcagtttcatgtgtagacgagtatg		
**KanF**	gcaggcgagaaaggagag	Amplification of kanamycin (Template BKK ykuJ from Dr. Rebecca Hinrichs)	RS082_KAN
**KanR**	cgaggctcctgtcactgc		

Supplemental references

1. Konkol, M. A., Blair, K. M., and Kearns, D. B. (2013) Plasmid-encoded ComI inhibits competence in the ancestral 3610 strain of Bacillus subtilis *J Bacteriol* **195**, 4085-4093 10.1128/JB.00696-13
